# ACTM-838, a novel systemically delivered bacterial immunotherapy that enriches in solid tumors and delivers IL-15/IL-15Rα and STING payloads to engage innate and adaptive immunity in the TME and enable a durable anti-tumor immune response

**DOI:** 10.18632/oncotarget.28769

**Published:** 2025-10-06

**Authors:** Kyle R. Cron, Ping Fang, Oanh Pham, Julie Janes, John Brandenburg, William Lu, Jonathan Zhu, Bret Peterson, Sara Tribble, Haixing Kehoe, Anastasia Makarova, Alex Iannello, Jean Chan, Justin Skoble, Hailey He, Chris Rae, Christopher D. Thanos, Akshata R. Udyavar

**Affiliations:** ^1^Actym Therapeutics, Berkeley, CA 94710, USA

**Keywords:** tumor microenvironment, bacterial vector, myeloid cells, STING, IL-15

## Abstract

STACT is a modular, genetically engineered live attenuated *S.* Typhimurium bacterial platform that enables tissue-specific localization and cell-targeted delivery of large, multiplexed payloads via systemic administration. It has been engineered to minimize systemic toxicity and to enrich in the tumor microenvironment (TME) via metabolic dependency and showed a decreased systemic inflammatory cytokine profile compared to its parent strain VNP20009. ACTM-838 utilizes the STACT platform to deliver IL-15/IL15Rα and a constitutively active STING to tumor-resident phagocytic antigen-presenting cells. Upon intravenous (IV) dosing to tumor-bearing mice, ACTM-838 distributed and enriched in the TME, exhibited specific uptake in tumor-resident phagocytic cells and led to expression of human IL-15/IL15Rα and murine IFNα in the tumor. ACTM-838 induced comprehensive TME changes to an immune permissive anti-tumor phenotype with a decrease in exhausted T-cells and Tregs and an increase in cytolytic T-cells and MHCII-high proliferating myeloid cells. ACTM-838-treated tumors exhibited upregulated anti-tumor innate and adaptive immunity expression profiles, T-, NK- and B-cell infiltration and downregulated cell cycle, DNA damage and TGFβ responses. Single-cell RNAseq and flow cytometry data confirmed activation and infiltration of both innate and adaptive immune cells. ACTM-838 showed durable anti-tumor efficacy in multiple murine tumor models and synergized with anti-PD1 therapy in combination.

## INTRODUCTION

Immune checkpoint blockade (ICB) therapies represent a significant advance in cancer treatment, but only a minority of patients with cancer exhibit durable responses [1]. Rational combinations have boosted responses, but there remains a high unmet need to identify combination therapies that can maximally potentiate an anti-tumor response while minimizing toxicity [2–4]. The effectiveness of T-cell-targeted ICB relies on the presence of pre-existing anti-tumor T-cells within the TME often referred to as “hot tumors”. Not all patients with high T-cell content, tumor mutation burden or PDL1 expression respond well to ICB, and many patients eventually develop resistance [4, 5]. One mechanism of resistance is the increased infiltration of myeloid and stromal cells leading to secretion of immunosuppressive cytokines and expression of adenosine-generating enzymes and receptors, which promotes tumor progression and T-cell suppression [6]. Thus, it is important to engage and reprogram immunosuppressive innate myeloid cells for antigen presentation and T-cell priming and create an immune-permissive TME to achieve a sustained anti-tumor immune response. A key challenge, though, is that the levels of immune modulatory proteins needed to appropriately shift the TME and engage innate immunity are higher than the levels that can be tolerated systemically [7–10]. Early efforts to address this challenge have included intra-tumoral delivery of immune modulatory therapies (e.g., cytokines or inducers of cytokines), but many have failed to generate abscopal responses [11, 12]. This suggests the need for systemically administered but locally acting TME modulating agents, including gene delivery therapies. To date, systemically administered gene delivery therapies have been challenged by several factors: limited tissue-targeted delivery beyond the liver, limited payload carrying capacity, difficulty of controlling activity once delivered, as well as complex manufacturing [13]. An ideal anti-tumor strategy would be a systemically administered gene delivery vector that localizes and delivers therapeutic payloads to the TME, such that the immune modulatory therapies act locally within tumors throughout the body.

Examples of immune modulatory cytokine therapies that exhibit lack of tolerability with systemic administration and require localized delivery include stimulator of interferon genes (STING)-induced type I interferons (IFN) and interleukin-15 (IL-15). STING is an intracellular transmembrane receptor expressed in the endoplasmic reticulum of both immune and non-immune cells that facilitates innate immune signaling. In response to the presence of bacteria or intracellular cytoplasmic DNA, STING is activated leading to the transcription of proinflammatory cytokines such as type I IFNs (IFNα/β), IL-6, TNFα and chemokines (CCL5, CXCL9/10/11). Type I IFN-mediated transcription in the TME results in maturation, migration, antigen-presentation and activation of myeloid cells, T-cells and NK-cells. STING also generates anti-tumor immunity in a type I IFN independent manner, collectively facilitating the transformation of an immune suppressive (pro-tumor) to an immune permissive (anti-tumor) TME [14]. Of note, recent studies suggest it is optimal to engage STING-mediated pathways within APCs in the TME [15]. Several STING agonists have been studied in clinical trials where intra-tumoral delivery resulted in tumor regression of injected lesions, but no regressions were observed in non-injected lesions suggesting absence of a systemic anti-tumor effect [16].

IL-15 is required for the development and homeostasis of memory CD8 T-cells and NK-cells. IL-15 can be produced by multiple cell types such as monocytes, macrophages, dendritic cells (DCs), B-cells, T-cells, endothelial and stromal cells. IL-15 primarily functions in a cell-cell contact-dependent manner via trans-presentation of membrane-bound IL-15/IL-15Rα heterodimer and does not appear to circulate in blood without its receptor due to its high affinity for IL-15Rα. IL-15 and IL-15Rα appear to require surface co-expression to be functional, with eventual cleavage and secretion of the bioactive heterodimer *in vivo*; whereas, the single chain IL-15 is poorly secreted and unstable [17, 18]. Multiple clinical approaches to deliver CAR-T/NKs or DCs incorporate IL-15 biology because IL-15 has been shown to be important for the sustained activation of these cell therapies *in vivo* [19]. While systemically delivered IL-15 has been challenging, the first locally delivered IL-15/IL-15Rα therapy, ANKTIVA^®^, was recently approved in combination with Bacillus Calmette-Guerin for non-muscle invasive bladder cancer [20].

Prior to the advent of chemotherapy, Coley’s toxin, a mixture of heat-inactivated bacteria *Streptococcus pyogenes* and *Serratia marcescens*, was used to treat more than a thousand patients with cancer over 40 years with several hundred achieving near complete regression [21, 22]. Recently, bacteria such as *Clostridium*, *Listeria*, *E.coli* and *Salmonella* have been shown to be naturally capable of homing to tumors, proliferating locally in the TME and eliciting strong anti-tumor responses when systemically administered [23]. The Gram negative facultative anaerobic bacterium *S.* Typhimurium is a cancer immunotherapeutic platform with several advantages including (a) its ability to grow in both aerobic and anerobic conditions, (b) its ability to be engineered for metabolic dependencies found in the TME, (c) its ability to carry plasmids with large genetic payloads to the tumor milieu and tumor-resident APCs, (d) its sensitivity to antibiotics for an off-switch, and (e) its ease of manufacturing [23, 24]. Several groups have exploited the inherent tumor specificity of *S.* Typhimurium to deliver disease-modifying payloads to the tumor milieu to mediate anti-tumor effects. Examples include the delivery of L-methioninase [25], tumor antigens [26] and IL-15 [27].

A key challenge in utilizing bacteria as a gene delivery vector is the possibility for an excessive host inflammatory response to bacterial pathogen-associated molecular patterns (PAMPs) via toll-like receptors (TLRs). Response to wild-type (WT) *S.* Typhimurium is largely mediated through TLR2 (lipoproteins), TLR4 (LPS) and TLR5 (flagellin) signaling, which activates NFκB and inflammasome pathways, resulting in production of pro-inflammatory cytokines, such as TNF-α, IL-6, IFNγ, IL-1β and IL-18 [28]. Thus, engineering *S.* Typhimurium for effective anti-tumor immunity includes preventing toxic pro-inflammatory responses in the periphery.

The *S.* Typhimurium Attenuated Cancer Therapy (STACT) platform was created from the parental strain VNP20009 through genome engineering to improve safety for systemic administration and eliminate immunosuppressive factors impacting CD8^+^ T-cell responses. This was achieved by attenuating TLR-mediated systemic cytokine production thought to be associated with dose-limiting toxicities (DLTs) in patients treated with VNP20009 [29]. STACT has been modified to be a live, programmable, well-tolerated, systemically delivered immunotherapy, where genes regulating expression and/or characteristics of LPS, flagella, curli fimbriae and asparaginase-II were deleted, resulting in a non-pathogenic microbe. STACT was designed to carry plasmids (Supplementary Figure 1H) that encode various payloads (RNA, peptides, proteins and gene-editing effectors) to increase therapeutic efficacy beyond the effects of the bacterial chassis itself. To this end, ACTM-838 is a STACT chassis that harbors DNA plasmids encoding two genetic payloads driven by a CMV promoter: (1) a single-chain fusion protein of the IL-15Rα sushi domain linked to the IL-15 cytokine (IL-15plex) [30]; and (2) a constitutively active version of STING with 2 gain-of-function mutations and a Tasmanian C-terminal domain to increase type I IFN activity and reduce NF-κB-mediated IL-6 secretion [31, 32] (eSTING) (diagrammed in Supplementary Figure 1G). ACTM-838 was engineered to not require antibiotic-resistance cassettes for plasmid maintenance, rendering it sensitive to frontline antibiotics and providing an off-switch in the clinic if needed. Plasmid maintenance was accomplished by removing an essential gene (*asd*) from the bacterial genome and inserting it on the plasmid. Of note, the human payloads in ACTM-838 are species cross-reactive, precluding the need to use a surrogate drug in murine studies.

Here we describe the validation of ACTM-838 in delivering the immune-modulatory payloads IL-15plex and eSTING to phagocytic myeloid cells in solid tumors after systemic administration and elucidate its mechanism of action in generating durable anti-tumor immunity in murine syngeneic tumor models.

## RESULTS

### Enhanced safety and sustained tumor colonization of ACTM-838 enables functional secreted IL15plex and eSTING-mediated IFNβ activity in human target cells

The STACT platform was developed from the parental *S.* Typhimurium VNP20009 strain via genomic engineering to eliminate bacterial-mediated T-cell immunosuppressive factors and to reduce TLR-mediated systemic cytokine responses associated with DLTs in subjects treated with VNP20009 [29]. Extensive details on the strain engineering are described in Supplementary Data and Supplementary Table 1. Briefly, STACT was engineered with deletions in genes encoding the flagellum, curli fimbriae, L-asparaginase and modifiers of LPS acylation as well as antibiotic sensitivity in the clinic (Supplementary Tables 1 and 2). The flagellum is an external structure that enables invasion of epithelial and endothelial cells, allows for Salmonella immune escape and is a TLR5 agonist (Supplementary Figure 1A) [33]. Thus, absence of flagella adds cell-type specificity to STACT wherein it is only taken up by phagocytic APCs and not epithelial or endothelial cells and rapidly destroyed by M2 macrophages once internalized (Supplementary Figure 1B, 1C). Deletion of genes involved in the curli fimbriae (TLR2 agonist) in STACT prevented the formation of biofilms, further improving safety [34] (Supplementary Figure 1D). Like VNP20009 [35], STACT is also a purine auxotroph and is unable to grow without purine/adenine supplementation (Supplementary Figure 1E), allowing for tumor-specific enrichment in the purine-rich TME. VNP20009 expresses both penta- and hexa-acylated LPS, the latter of which is more inflammatory in humans, via TLR4 activation [35, 36]. To attenuate systemic IL-6 responses, the STACT genome was further modified to express only penta-acylated LPS (Supplementary Figure 1F). To further improve on VNP20009, we deleted the gene encoding L-asparaginase, which inhibits the T-cell response by depleting local L-asparagine concentration in the TME [37]. With the engineered asparaginase gene deletion, STACT lacked the ability to convert extracellular L-asparagine to aspartic acid, resulting in restored TCRβ expression in T-cells and cytokine secretion (Supplementary Figure 1I, 1J).

**Figure 1:**
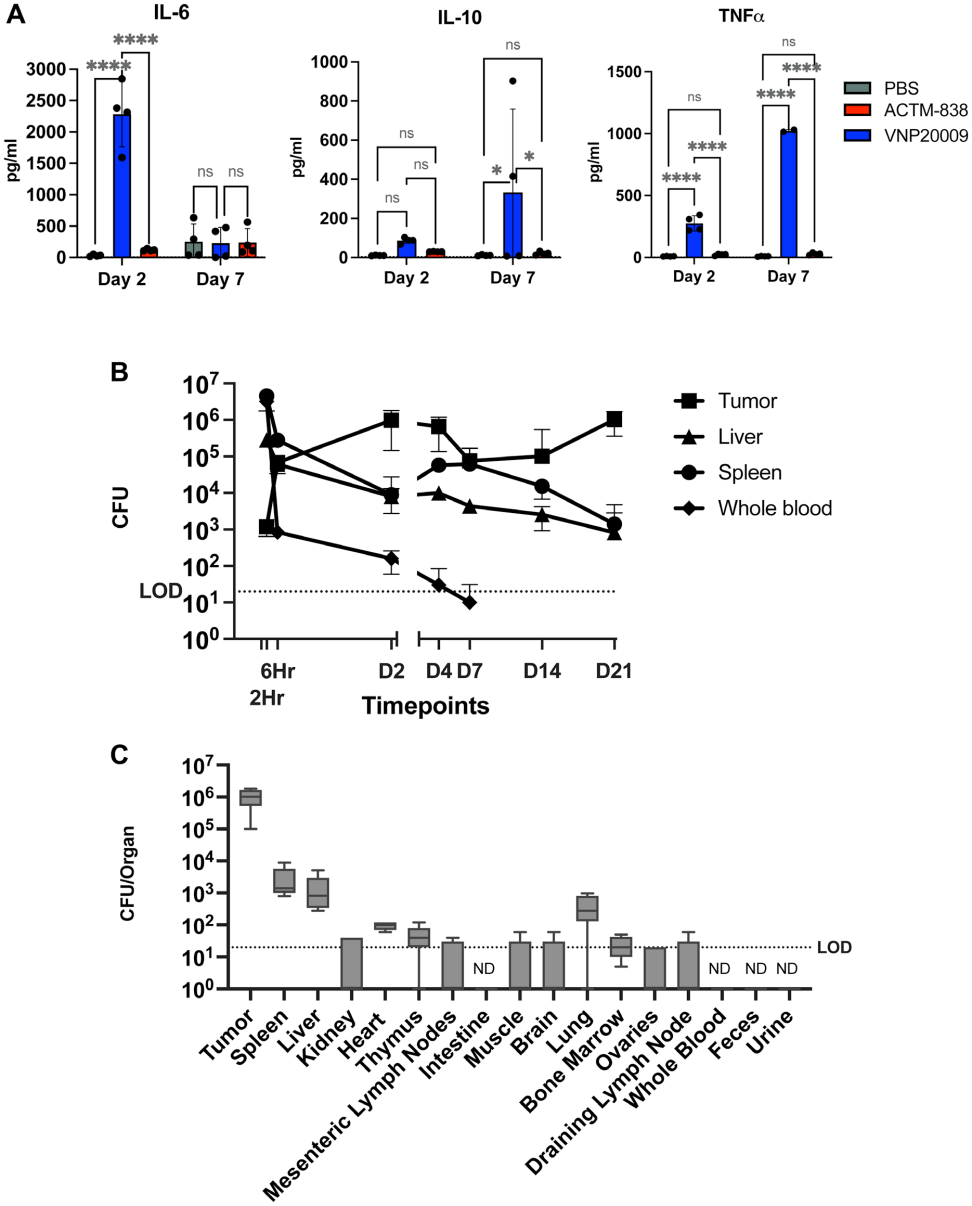
Modified STACT chassis of ACTM-838 increases safety and maintains enriched tumor colonization with a single IV dose. (**A**) ACTM-838 exhibits significantly reduced systemic cytokines compared to VNP20009 in EMT6-tumor bearing BALB/c mice on days 2 and 7 post IV dosing (3e7 CFU/mouse). Dotted line indicates LOD for given cytokine. (**B**) Tissue biodistribution kinetics in tumor, liver, spleen and blood over time in EMT6-tumor bearing mice dosed with 3.8e7 CFU/mouse of ACTM-838 (*n* = 5 per timepoint). LOD for all tissues is 20 CFU/organ and whole blood is 10 CFU/mL. (**C**) Blood levels, urine/fecal shedding and tissue biodistribution on day 21 post dosing of ACTM-838 (3.8e7 CFU/mouse) (*n* = 5). Bone marrow, blood and urine are denoting CFU/mL levels, other tissues denote CFU/organ. ACTM-838 shows high tumor colonization compared to other healthy tissues and blood. Data are expressed as median ± standard deviation (SD) per organ (*N* = 5 per group). Statistical significance was determined using two-way ANOVA followed by Tukey’s multiple comparisons test. ^*^ *p* < 0.05; ^**^ *p* < 0.01; ^***^ *p* < 0.0001; ^****^ *p* < 0.00001. Abbreviations: CFU: colony-forming unit; LOD: limit of detection; ND: not detected; pg/ml, picogram/milliliter.

To test if the genomic modifications in STACT increase safety, we assessed systemic cytokine responses on days 2 and 7 after a single IV dose of either VNP20009 or ACTM-838 in EMT6 triple negative breast cancer (TNBC) tumor-bearing mice. IL-2 was very low or below LOD in most conditions (Supplementary Figure 2). Compared to VNP20009, treatment with ACTM-838 resulted in significantly lower levels of cytokines activated by TLR2 (IL-1β, IL-10), TLR4/9 (IL-6, IL-10, TNFα) as well as chemokines (CXCL1/10, CCL2), suggesting that the genomic modifications in STACT dampen the proinflammatory cytokine response in the periphery (Figure 1A; Supplementary Figure 2). We further tested the tolerability via systemic IV administration in female C57Bl/6 mice which exhibited a 50-fold better tolerability of ACTM-838 compared to VNP20009 (discussed in the Supplementary Text).

To understand the impact of genomic modifications on tumor colonization and biodistribution kinetics, EMT6 orthotopic tumor-bearing mice were dosed intravenously (IV) with a single dose of ACTM-838 at 3.8e7 CFU/mouse. Organs and tissues were collected, processed, and live CFU plating was performed at various timepoints to measure colonization. Rapid biodistribution of ACTM-838 was observed in several tissues within 6 hours and enrichment in tumors was observed within 24 hours (Figure 1B; Supplementary Figure 3; Supplementary Table 3). Other organs showed rapid decline of ACTM-838 colonies over time while tumors exhibited persistent colonization. Importantly, ACTM-838 was not detected in the urine or feces of EMT6 tumor-bearing BALB/c mice at any timepoint, suggesting the bacterial drug has a low risk of environmental impact or transmission. In addition, ACTM-838 was cleared from blood within 4 days of IV dosing. Despite the attenuating genomic modifications, ACTM-838 continued to show enriched tumor colonization at 1000-fold higher levels at day 21 as compared to spleen and liver, which are the natural sites for *Salmonella* clearance, and colonization was either not detectable or detectable at just above LOD in all other tested tissues (Figure 1C, Supplementary Table 3). Consistent with this biodistribution data in tumor-burdened mice, there were no major histopathological findings in IND-enabling non-human primate toxicology studies (data not shown).

The specificity of payload expression and activity was evaluated in human cells - HEK293 and THP-1 macrophages (Supplementary Data and Methods; Supplementary Figure 4). Briefly, a dose-dependent increase of IL-15plex secretion and eSTING-mediated IFN-β reporter activity was observed with ACTM-838 in HEK293-Dual-Null cells, a cell line with two integrated reporter genes induced by interferon response, (Supplementary Figure 4A–4D), suggesting that the ACTM-838 STACT chassis can effectively deliver its plasmids once internalized and enable payload expression and activity in target mammalian cells. Bacteria in media absent of HEK293T cells did not result in detectable IL-15, demonstrating that ACTM-838 payload is delivered to and expressed by mammalian cells (Supplementary Figure 4E).

To test the mechanism of uptake and payload expression in human macrophages, phagocytic activity was evaluated in THP-1-Dual cells (Supplementary Methods). Phagosomal internalization was measured 90 minutes post-treatment with pHrodo-labeled ACTM-838 which fluoresces in acidic compartments. Localization in phagolysosomes was indicated by pHrodo^+^ THP-1-Dual macrophages (Supplementary Figure 4F). ACTM-838 uptake levels were observed to be dose dependent in M0-, M1- and M2-like macrophages. After 48 hours, ACTM-838 treated macrophages showed a dose-dependent increase in eSTING-mediated IRF luciferase reporter activity which correlated with phagosomal uptake (Supplementary Figure 4G). To further confirm the phagocytic ACTM-838 uptake in human myeloid cells, healthy donor PBMCs were treated with pHRodo-labeled ACTM-838 and showed similar results where only cells capable of active phagocytosis such as monocytes, DCs and B-cells exhibited ACTM-838 uptake, while T-cells did not (Supplementary Figure 5).

### ACTM-838 induces pro-inflammatory antigen-presenting phenotypes in human macrophages

**Figure 2:**
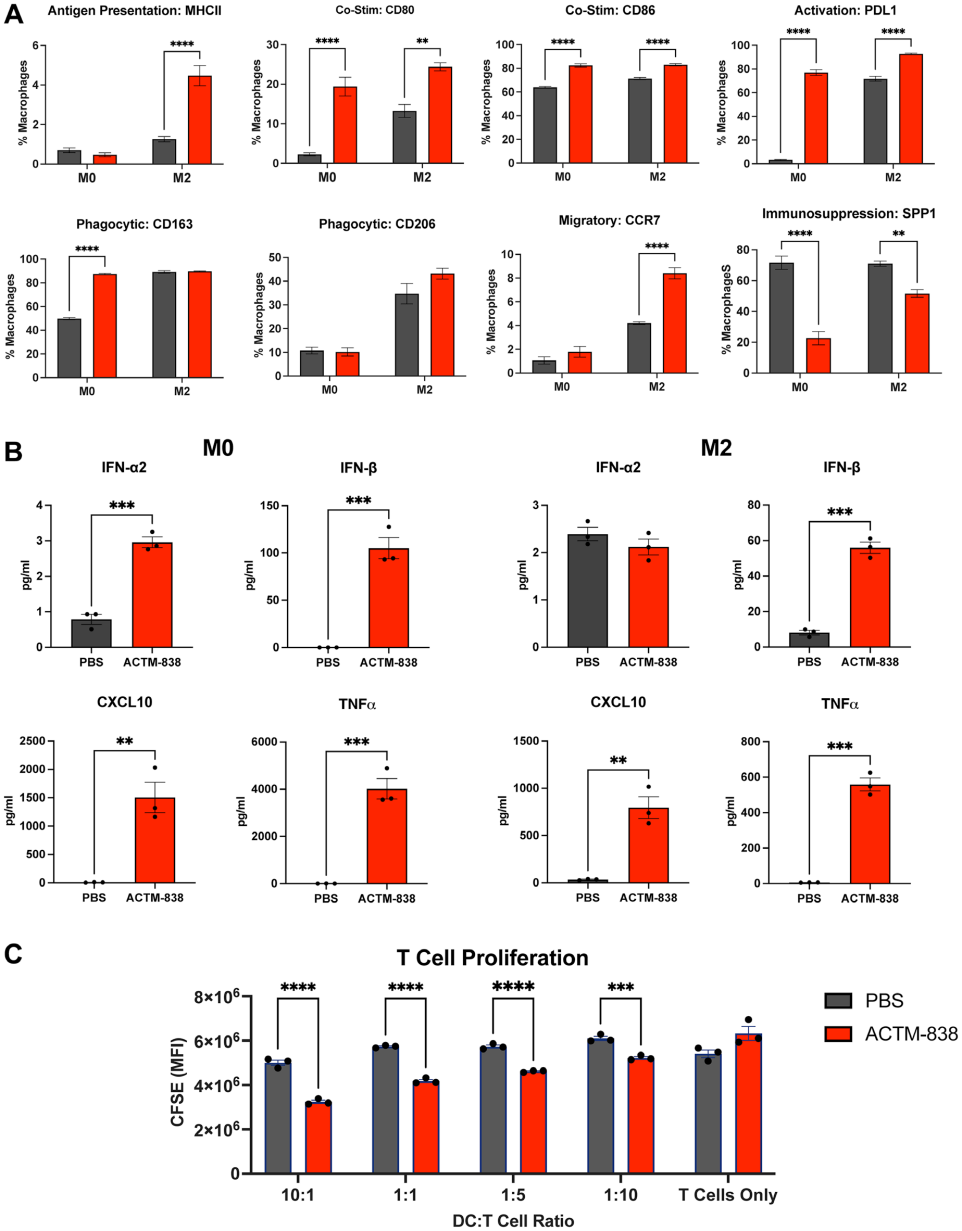
Human MDMs exhibit proinflammatory antigen presenting phenotype that can stimulate T-cell proliferation with ACTM-838 treatment. (**A**) Primary human MDMs were treated with MOI 40 of ACTM-838 or PBS for 1 hour. Cells were assessed for activation markers via flow cytometry at 48 hours. (**B**) Type I interferon-related cytokines detected in human macrophages 48 hours post-treatment with ACTM-838. (**C**) MLR assay shows significantly increased CD4 T-cell proliferation at 72 hours after co-culture with human moDCs (pre-treated for 48 hours with ACTM-838). T-cells were labeled with CFSE to detect cell proliferation via dye dilution. Data are presented as mean ± SEM analyzed by two-way ANOVA; ^*^ *p* < 0.05; ^**^ *p* < 0.01; ^***^ *p* < 0.0001; ^****^ *p* < 0.00001. Abbreviations: CD: cluster of differentiation; MHC: major histocompatibility complex; PBS: phosphate buffered saline; PDL1: Programmed death-ligand 1; IFN: interferon; MDM: monocyte derived macrophages; MOI: multiplicity of infection; TNF: tumor necrosis factor; CFSE: carboxyfluorescein succinimidyl ester; moDCs: monocyte-derived DCs.

To assess effects of ACTM-838 on primary human macrophages, human monocyte-derived macrophages (MDMs) were treated with ACTM-838 or PBS. At 48 hours post-treatment, an elevation in anti-tumor macrophage activation markers was observed, with increases in expression of MHCII, co-stimulatory markers CD86 and CD80, PDL1 and the migratory marker CCR7 (Figure 2A). Phagocytic markers CD163 and CD206 showed no change (except in M0-like MDMs) compared with PBS. Interestingly, CD206 was co-expressed in CD80 positive proinflammatory M1-like MDMs, suggesting a hybrid phenotype with pro-inflammatory and phagocytic properties (data not shown). CD206^+^CD80^+^ macrophages have been previously shown to be able to cross-present and activate antigen-specific T-cells in mice [38]. Also, SPP1, a marker of immunosuppressive macrophages [39], was decreased in ACTM-838 treated MDMs. ACTM-838 treatment also increased the secretion of several type I interferon pathway cytokines (IFN-α/β, CXCL10) and TNFα (Figure 2B), consistent with eSTING-pathway activity.

To assess T-cell activation mediated by ACTM-838, we treated human monocyte-derived dendritic cells (moDCs) with either PBS or ACTM-838 for 48 hours and then co-cultured different ratios of treated moDCs and CD4 T-cells. As a negative control, T-cells were treated directly with ACTM-838 or PBS in the absence of moDCs. ACTM-838 treated moDCs significantly increased CD4 T-cell proliferation at all effector-to-target ratios, suggesting ACTM-838-mediated myeloid activation can enhance T-cell proliferation and activation (Figure 2C).

Together, these data suggest ACTM-838 is rapidly internalized via phagocytosis leading to the activation of human macrophages into an activated anti-tumor phenotype, as indicated by phagocytic and antigen presentation markers.

### ACTM-838-mediated myeloid specific cellular internalization and TME-specific payload delivery *in vivo*

**Figure 3:**
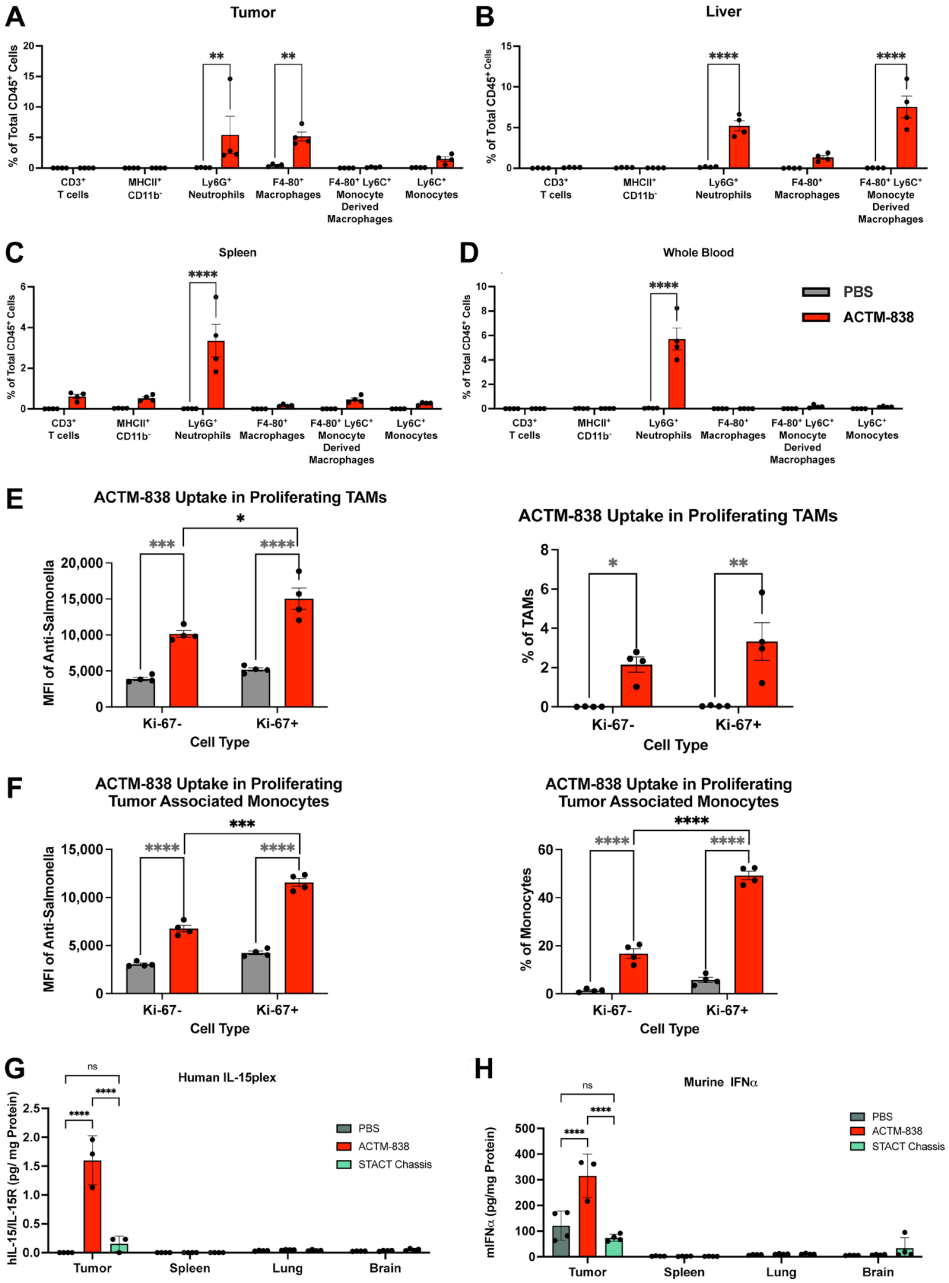
ACTM-838 exhibits myeloid specific cellular internalization and tumor-specific payload expression in EMT6-tumor-bearing mice. Internalization of ACTM-838 in phagocytic APC subsets within CD45^+^ immune population in EMT6tumor bearing BALB/c mice at 24 hours post treatment across tumor (**A**), liver (**B**), spleen (**C**) and whole blood (**D**). (**E**, **F**) Influence of myeloid proliferation on ACTM-838 uptake in the TME in EMT6 tumor-bearing BALB/c mice. (**G**, **H**) Human IL-15plex and murine IFNα (downstream target of eSTING) are only detected in the tumor and no other healthy tissues or blood in EMT6-Tumor Bearing BALB/c Mice at Day 7 post-ACTM-838 treatment. ^*^ *p* < 0.05; ^**^ *p* < 0.01; ^***^ *p* < 0.0001; ^****^ *p* < 0.00001. Abbreviations: CD: cluster of differentiation; PBS: phosphate buffered saline; IFN: interferon; pg/ml, picogram/milliliter.

To assess the internalization of ACTM-838 *in vivo*, EMT6-tumor bearing mice were administered a single IV dose (6e7 CFU/mouse) of ACTM-838. At 24 hours post-treatment, ACTM-838 uptake was observed specifically in phagocytic APCs such as monocytes, macrophages and neutrophils in the tumor tissue and liver, and neutrophils in the spleen and whole blood (Figure 3A–3D). Importantly, ACTM-838 showed no or minimal internalization in the CD45^-^ population comprising of epithelial and endothelial cells and fibroblasts (Supplementary Figure 6A), consistent with the cell type specificity observed *in vitro* (Supplementary Figure 1B and Supplementary Figure 5). Minimal or no uptake (<1%) was observed on day 7 in the spleen, liver or whole blood, while the tumor continued to show uptake in the myeloid cells (Supplementary Figure 6B, 6C). This is consistent with high ACTM-838 TME colonization and low or no colonization in liver and blood over time (Figure 1B, 1C). Interestingly, proliferating myeloid cells in the TME exhibited significantly higher ACTM-838 uptake, likely leading to higher levels of payload delivery and expression due to being in active cell division [39, 40] (Figure 3E, 3F).

To evaluate the ability of ACTM-838 to specifically deliver human IL-15plex and eSTING to the TME *in vivo*, EMT6-tumor-bearing mice were dosed with either PBS, ACTM-838 (3e7 CFU/mouse) or STACT chassis lacking active payload (3e7 CFU/mouse) and tissue lysates were evaluated for payload expression. Human IL-15plex and murine IFNα (eSTING pathway readout) protein expression were only observed in primary tumor and not in the lung, spleen or brain of mice receiving ACTM-838 (Figure 3G, 3H). The STACT chassis control strain did not lead to payload expression in any tested tissues, suggesting that payload delivery was ACTM-838-specific. These data suggest that ACTM-838 exhibits tumor-specific payload delivery *in vivo*, likely via the proliferating tumor-associated macrophages (TAMs), neutrophils and monocytes in the TME (Figure 3A, 3E, 3F).

### ACTM-838 confers durable anti-tumor efficacy as a single agent and synergistic anti-PD1 combination efficacy across multiple mouse models

**Figure 4:**
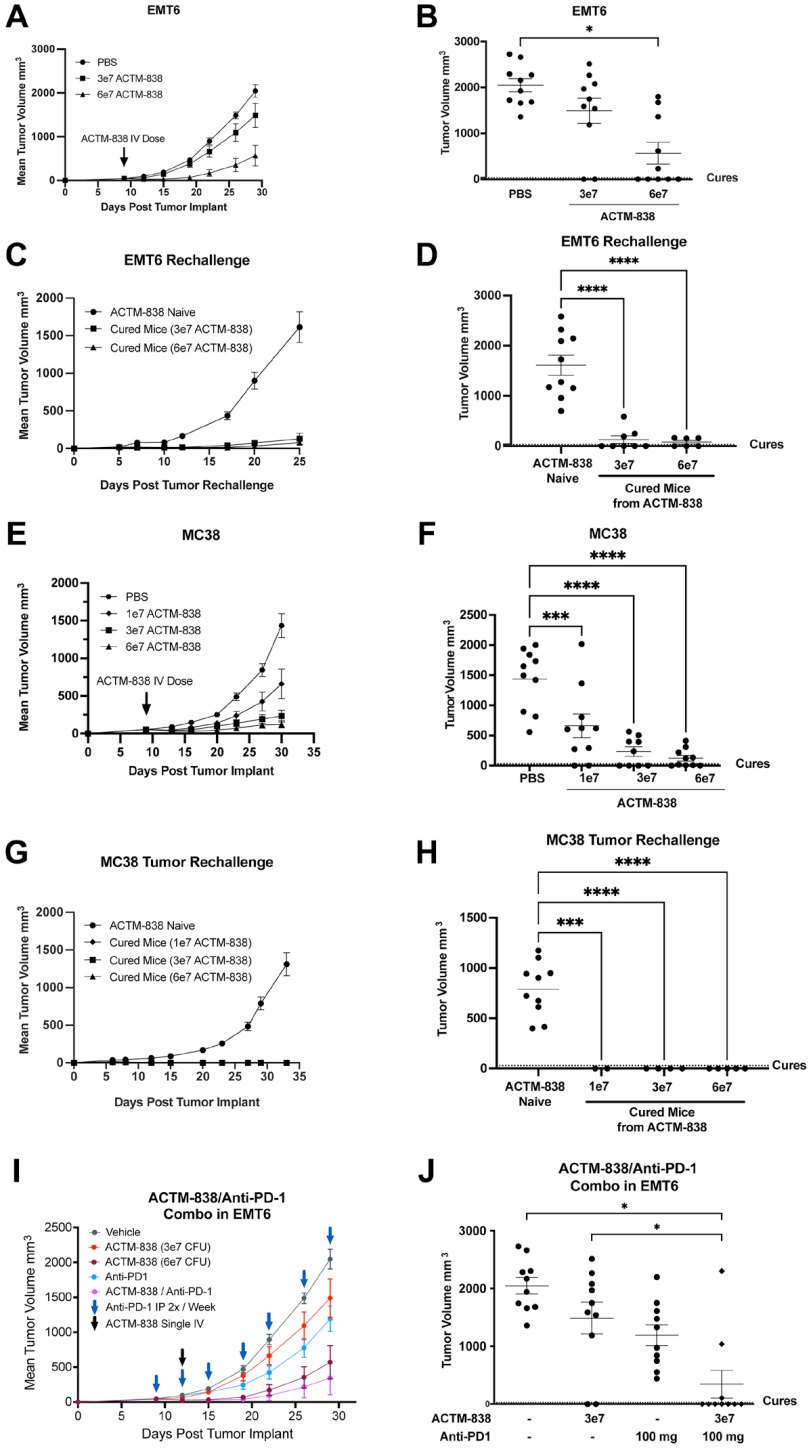
ACTM-838 shows dose dependent single agent efficacy and durable anti-tumor immunity across multiple tumor models. (**A**–**D**) Durable ACTM-838 single agent anti-tumor efficacy in immune excluded, immune checkpoint blockade refractory EMT6 orthotopic TNBC model. Dose response efficacy (*n* = 10 per condition) (A), tumor volumes at end point (B). (C, D) show tumor volumes over time and at endpoint with tumor rechallenge on contralateral mammary fat pad in ACTM-838-cured animals after 30 days in remission and at day 25 post-rechallenge respectively (ACTM-838 Naïve *n* = 10, Cured Mice 3e7 CFU/mouse *n* = 8, Cured Mice 6e7 CFU/mouse *n* = 6). (**E**–**H**) Durable ACTM-838 single agent anti-tumor efficacy in immune inflamed, immune checkpoint blockade sensitive MC38 subcutaneous colon tumor model showing dose response efficacy and tumor volumes at end point (*n* = 10 per condition). (**G**, **H**) show tumor volumes over time and at endpoint with tumor rechallenge on contralateral flank in ACTM-838-cured MC38 animals after 30 days in remission and at day 35 post-rechallenge respectively (ACTM-838 Naïve *n* = 10, Cured Mice 1e7 CFU/mouse *n* = 2, Cured Mice 3e7 CFU/mouse *n* = 4, Cured Mice 6e7 CFU/mouse *n* = 5). Data are expressed as mean ± SEM using ANOVA and Tukey’s multiple comparison test. ^*^ *p* < 0.05; ^**^ *p* < 0.01; ^***^ *p* < 0.0001; ^****^ *p* < 0.00001. (**I**, **J**) ACTM-838 at 3e7 CFU/mouse shows synergistic combination efficacy with anti-PD1 with durable remissions in EMT6 tumor bearing mice over time and at endpoint (Vehicle *n* = 10, ACTM-838 3e7 CFU/mouse *n* = 12, ACTM-838 6e7 CFU/mouse *n* = 11, Anti-PD1 *n* = 10, ACTM-838/Anti-PD-1 *n* = 10). Data are expressed tumor volume (mm^3^) ± standard error of the mean (SEM). ^*^ *p* < 0.001 (Benjamini and Hochberg multiple comparisons test).

Given the myeloid-specific cellular uptake and payload expression in the TME, we next assessed *in vivo* efficacy of ACTM-838 administered as a single IV dose in the EMT6 orthotopic model. EMT6 is an immune checkpoint blockade-refractory TNBC syngeneic tumor model that has been shown to exhibit an immune-excluded phenotype where T-cells are excluded to the periphery of the tumor [40]. A dose-dependent and durable anti-tumor effect was observed with a higher percentage of cures in mice receiving the higher dose (20% and 50% with 3e7 and 6e7 CFU/mouse respectively) (Figure 4A, 4B; chassis-alone effects in Supplementary Figure 7). Mice that achieved complete cures following the initial treatment continued to remain in remission for 30 days and were subsequently re-challenged with fresh EMT6 tumor cells on the contralateral mammary fat pad. 50-70% of the animals remained in remission after being rechallenged with fresh tumor cells, demonstrating durable anti-tumor immunity (Figure 4C, 4D). We further confirmed the anti-tumor memory response by using an anti-CD8β antibody to deplete CD8 T-cells before tumor rechallenge. The non-depleted control cured animals remained in remission, whereas the CD8-T-cell-depleted animals exhibited tumor growth (Supplementary Figure 8A–8C), suggesting the durability of response is likely mediated by CD8 T-cells.

Next, we validated ACTM-838 dose response efficacy in an immune-inflamed, immune checkpoint blockade sensitive, subcutaneous MC38 syngeneic colon tumor model [41]. Similar to the results in the EMT6 model, dose-dependent tumor growth inhibition and complete cures at higher doses of ACTM-838 (20, 40 and 50% cures at 1e7, 3e7, and 6e7 CFU/mouse respectively) were observed (Figure 4E, 4F). After 30 days in remission post-ACTM-838 dosing, we rechallenged the cured mice by implanting fresh MC38 tumor cells in the contralateral flank. 100% of the cured animals remained in remission for 30 days after rechallenge demonstrating durable anti-tumor immunity (Figure 4G, 4H). Of note, the EMT6 and MC38 syngeneic models are on the BALB/c and C57BL/6 backgrounds, respectively, suggesting that ACTM-838 is effective in multiple genetic backgrounds.

The mammary-specific polyomavirus middle T antigen overexpression mouse model (MMTV-PyMT) is a spontaneously metastasizing genetically engineered mouse model (GEMM), with molecular and histological resemblance to human metastatic breast cancer [42]. Metastatic tumors present a challenge for therapeutic approaches due to immune composition and TME differences between the primary and metastatic lesions. We hypothesized that ACTM-838 being dependent on purines for its survival and proliferation, should be able to colonize metastatic lesions due to high adenosine levels in the metastatic TME [43]. To assess ACTM-838 efficacy in MMTV-PyMT GEMM, 6–8-week-old female mice were administered a single IV dose of ACTM-838 (6e7 CFU/mouse) or vehicle control. ACTM-838 treatment significantly reduced the cumulative tumor volume at endpoint (Supplementary Figure 8D). ACTM-838 as a single agent was effective in reducing the number of spontaneous tumors developing over time, where each control animal developed a maximum of 10 tumors while ACTM-838 treated animals only developed an average of 6 tumors (Supplementary Figure 8E). In addition, ACTM-838 reduced spontaneous lung metastasis in this model (Supplementary Figure 8F). Together these data suggest an overall impact of ACTM-838 on delaying tumor progression and metastasis.

To assess ACTM-838 in a second metastatic model, we used a lung metastasis tail vein injection model of EMT6. ACTM-838 treatment (6e7 CFU/mouse) demonstrated a significant reduction in metastatic lung lesions compared to PBS control (Supplementary Figure 8G). This efficacy correlated with a significant increase in IL-15plex protein levels in the lung which were not detected in the spleen or serum, indicating the expression of ACTM-838 delivered payloads specifically in the lung metastases (Supplementary Figure 8H). Overall, these data demonstrate the efficacy of ACTM-838 and payload delivery to primary and metastatic tumor sites.

PDL1 expression is upregulated on activated myeloid cells to prevent excessive inflammation and is associated with favorable responses to anti-PD1 therapy [44]. Anti-PD1 is standard-of-care in multiple solid tumor indications; however, a majority of tumors are resistant due to multiple mechanisms [45]. ACTM-838 treatment upregulated PDL1 expression in macrophages (Figure 2A). To assess ACTM-838 synergy with immune checkpoint blockade therapies, we conducted *in vivo* studies of ACTM-838 in combination with anti-PD1 in immune checkpoint blockade refractory (EMT6) and sensitive (MC38) models. At a sub-optimal 3e7 CFU/mouse dose for the EMT-6 model, ACTM-838 monotherapy treatment demonstrated anti-tumor efficacy as indicated by a reduced tumor volume and 20% remissions compared with vehicle-treated controls; whereas anti-PD1 as a single agent did not exhibit any remissions. ACTM-838 at 3e7 CFU/mouse in combination with anti-PD1 demonstrated synergistic anti-tumor efficacy with improved remissions from 20% (ACTM-838 single agent) to 70% (in combination with anti-PD1) (Figure 4I, 4J). The combinations were well tolerated with no clinical signs and minimal reversible body weight loss (data not shown).

In the MC38 model, single agent treatment of ACTM-838 showed equivalent efficacy to anti-PD1 with similar number of durable remissions (40%) in this anti-PD1 responsive tumor model. ACTM-838 at 3e7 CFU/mouse in combination with anti-PD1 showed synergistic anti-tumor efficacy, with 100% cure rates (Supplementary Figure 9A, 9B). No significant body weight loss or death was observed in any treatment regimens of ACTM-838 alone or in combination with anti-PD1 in any of the syngeneic models (data not shown).

### Comprehensive activation of innate and adaptive immunity in the TME to enable a durable anti-tumor immune response

**Figure 5:**
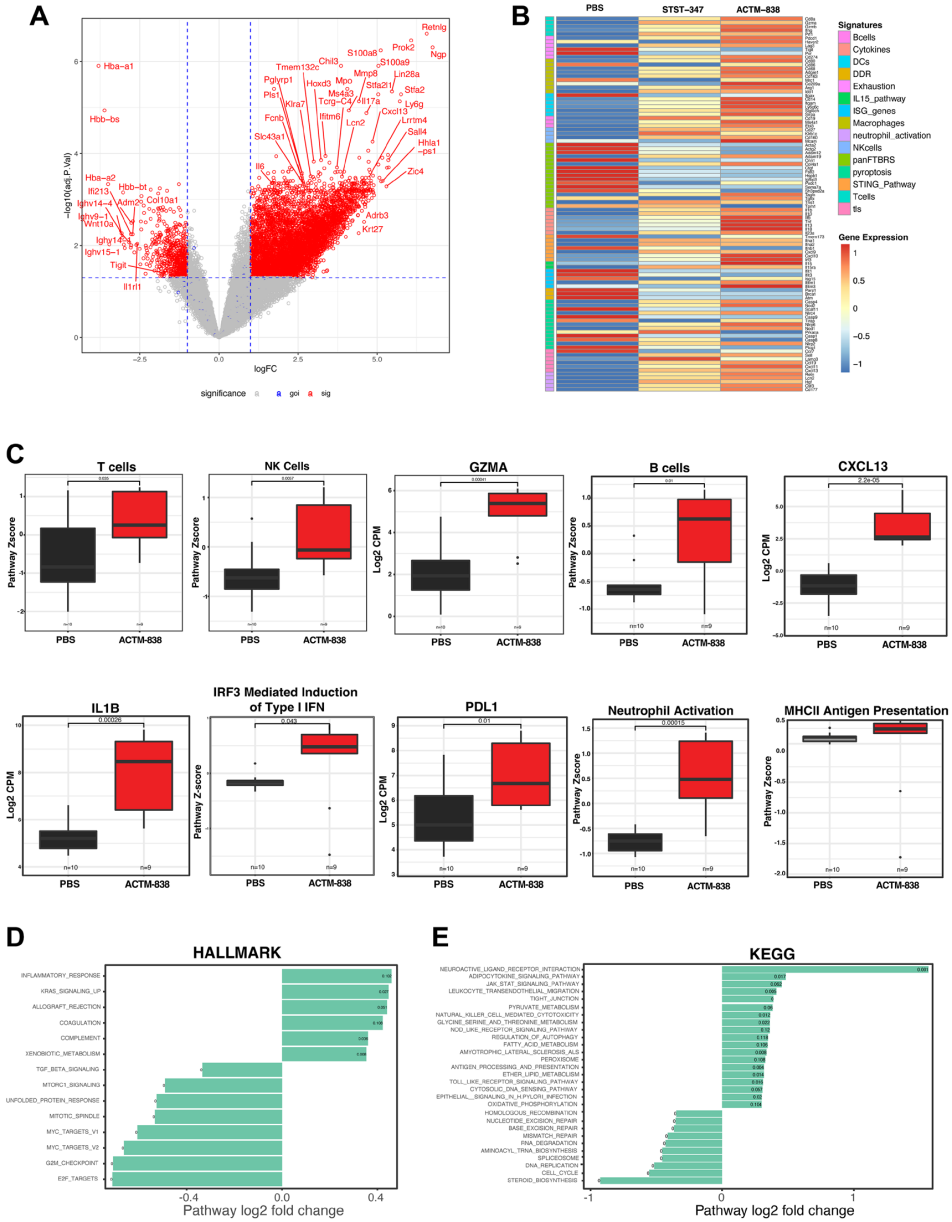
ACTM-838 comprehensively activates the immunosuppressive TME in EMT6 tumor bearing mice. (**A**) Differential expression analysis on bulk RNAseq comparing PBS vs. ACTM-838 treated EMT6 tumors on day 4 post-treatment (6e7 CFU/mouse). (**B**) Heatmap of genes representing different pathways of the innate and adaptive immune system. The STST-347 strain, delivering truncated and non-signaling payloads (IL-15 sushi domain without IL-15 cytokine and human STING without the STING C-terminal tail) was used as a STACT chassis control (PBS *n* = 10, STST-347 *n* = 5, ACTM-838 *n* = 5). (**C**, **D**) Differential gene set enrichment analysis (GSEA) with Hallmark and KEGG pathways comparing ACTM-838 vs. PBS showing upregulated inflammatory pathways in ACTM-838 treated tumors. (**E**) Boxplots denoting representative genes and pathway Z-scores.

ACTM-838 converts human macrophages to an anti-tumor antigen presenting phenotype with reduced SPP1 expression *in vitro* (Figure 2). To better understand the effects of the ACTM-838 delivered payloads on the TME, we performed bulk RNAseq on EMT6 tumors on day 4 after ACTM-838 treatment (6e7 CFU/mouse). Treated tumors exhibited broad changes in the TME with significantly upregulated genes related to adaptive immune lineages, such as increased B-cell (CD19/20) and T-cell infiltration (CD8α, CD3, TCRγ), as well as cytolytic activity (IFNγ, granzyme A/B, perforin) (Figure 5A–5C). ACTM-838 treated tumors also exhibited upregulated innate immune genes and signatures indicating infiltration and activation (MHC I/II antigen presentation, PDL1) of macrophage, NK, DC and neutrophils. In addition, cytokines (IL-6, TNFα, IL-1β, IL-17), chemokines (CXCL9/10/11/13), IL-15-related genes (IL-15, IL-15Rα) and STING-regulated interferon pathway (IFNα/β, IFITM1/3/6) were also upregulated in response to ACTM-838. In contrast, there was a decrease in T-cell exhaustion markers (TIGIT, CD155), stromal immunosuppressive signatures [40] and DNA damage response (DDR). Of note, modulation of the TME was observed, but to a lesser degree, when mice were treated with the STST-347 chassis control indicating that the STACT chassis and payloads may each have separate but additive impacts. (Figure 5B). Differential pathway gene set enrichment analysis (GSEA) showed significantly upregulated inflammatory response, complement, antigen processing and presentation, cytosolic DNA sensing, Jak-Stat and TLR signaling, autophagy, NK-cell cytotoxicity, leukocyte migration and metabolic pathways (Figure 5D, 5E). In contrast, significantly downregulated pathways included cell cycle related pathways (G2M checkpoint, mitotic spindle, cell cycle, DNA replication), TGFβ signaling, DDR pathways (homologous recombination, nucleotide, mismatch and base excision repair) and steroid biosynthesis pathways.

**Figure 6:**
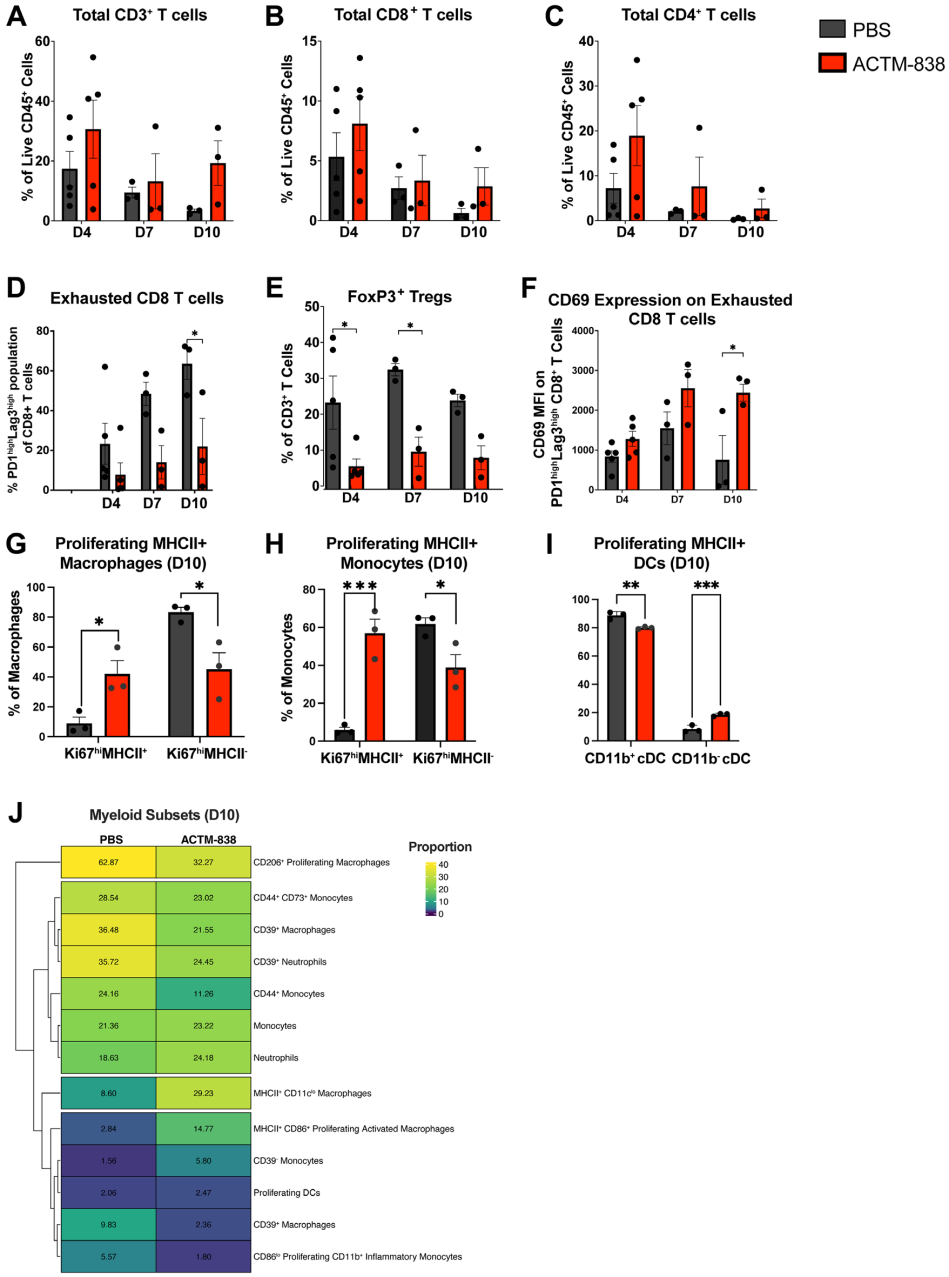
ACTM-838 treated EMT6 tumors exhibit increased activation and decreased immunosuppression across innate and adaptive immune subsets over time. (**A**–**C**) Of total CD45^+^ immune cells in PBS vs. ACTM-838 over time, increased proportions of total CD3^+^, CD8^+^ and CD4^+^ T-cells are observed with ACTM-838 treatment (6e7 CFU/mouse). (**D**) Exhausted (PD1^high^Lag3^high^) T-cells and (**E**) CD4^+^ Foxp3^+^ Tregs are significantly decreased over time with ACTM-838 treatment. (**F**) Activation marker CD69 significantly increases on exhausted CD8^+^ T-cells suggesting activation with ACTM-838 treatment. (**G**–**I**) Proliferating (Ki67^+^) MHCII^+^ antigen-presenting macrophages, monocytes and CD11b^-^ cDCs are significantly increased on day 10 post ACTM-838 treatment. Data are expressed as mean ± SEM using ANOVA and Tukey’s multiple comparison test. (**J**) Heatmap of myeloid subpopulations identified by UMAP analysis on the myeloid and general immune staining panels (PBS *n* = 3, ACTM-838 *n* = 3) (Supplementary Figure 10). Populations with proportions less than 1.5% across treatment groups were excluded. ^*^ *p* < 0.05; ^**^ *p* < 0.01; ^***^ *p* < 0.0001; ^****^ *p* < 0.00001.

Flow cytometry analysis also indicated ACTM-838 induced broad activation of the EMT6 TME modulating both lymphoid and myeloid compartments over time. ACTM-838 treated (3e7 CFU/mouse) tumors showed an increased infiltration of total CD3^+^, CD4^+^ and CD8^+^ T-cells over a 10-day time course (Figure 6A–6C). ACTM-838 exhibited a significant decrease in exhausted (PD1^high^Lag3^high^) CD8 T-cells and CD4^+^Foxp3^+^ Tregs over time (Figure 6D, 6E). Exhausted CD8^+^ T-cells had higher expression of the activation marker CD69 in ACTM-838 treated tumors (Figure 6F). In the myeloid compartment, ACTM-838 treatment significantly increased the proportion of proliferating MHCII^+^ tumor-associated monocytes, macrophages and CD11b^-^ cDCs (Figure 6G–6I). To better understand myeloid activation, we performed clustering analysis of day 10 populations which identified multiple distinct myeloid subsets (Supplementary Figure 10). Interestingly, ACTM-838 treatment increased proportions of monocytes, neutrophils, MHCII^+^ proliferating DCs and decreased CD206^+^ proliferating macrophages (Figure 6J). Importantly, MHCII^+^CD11c^low^ and MHCII^+^CD86^+^ proliferating F4/80^+^ macrophages increased 4-5-fold with ACTM-838 treatment. CD39 and CD73 are enzymes that convert ATP to AMP and adenosine respectively and high expression of these enzymes on immune cells leads to immunosuppression [43]. ACTM-838 reduced proportions of CD39^+^ macrophages and neutrophils as well as CD73^+^ monocytes (Figure 6J).

**Figure 7:**
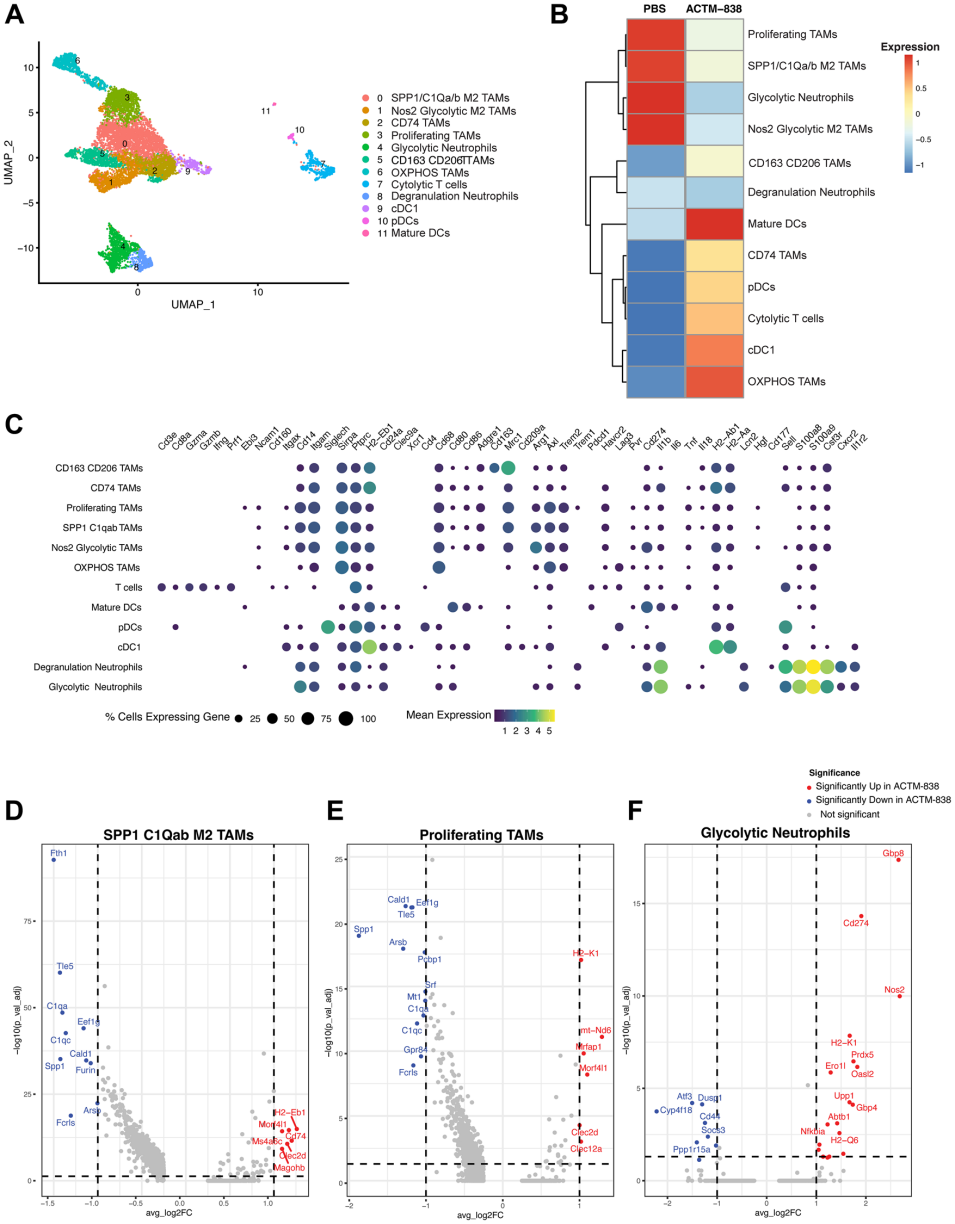
Single cell RNAseq analysis identifies novel myeloid subsets with ACTM-838 treatment. (**A**) UMAP analysis on single cell RNAseq data from ACTM-838 (6e7 CFU/mouse, *n* = 2) or PBS (*n* = 2) treated EMT6 tumors on day 7 post treatment with clusters annotated based on known cell type markers. (**B**) Heatmap showing mean proportions of immune subsets across PBS and ACTM-838 tumors (*N* = 2 per group). (**C**) Dotplot showing highly expressed representative markers for each subset. (**D**–**F**) Differential expression analysis in ACTM-838 vs. PBS treated tumors within SPP1 C1Qa/b M2 TAMs, proliferating Ki67^+^ TAMs and glycolytic neutrophils with significantly upregulated and downregulated genes in ACTM-838 denoted in red and blue respectively.

To further our understanding of the impact of ACTM-838 on the target myeloid cell types, we performed single cell RNAseq on EMT6 tumors 7 days post-treatment with either PBS or ACTM-838 (6e7 CFU/mouse) when tumors undergo remission. UMAP analysis focusing on the CD45^+^ immune cells identified 11 clusters (Figure 7A). Clusters were annotated based on expression of representative genes known in the literature (Figure 7C). ACTM-838 treatment induced an increase in cytolytic CD8 T-cells (expressing GZMA/B, IFNγ and perforin) (Figure 7B, 7C). Consistent with engagement of innate immunity, ACTM-838 treatment increased the proportion of cDC1 (Batf3^+^) [46], pDC [47, 48] and mature DCs with high expression of MHCII, CD80/86, IL-1β, and CD40, which are markers associated with DC maturation and T-cell priming [49, 50].

Different subsets of immunosuppressive TAMs (SPP1-C1Qa/b, proliferating, Nos2 glycolytic and OXPHOS TAMs) exhibited high expression of Trem2, Axl, Tim3 and Arg1, known markers of a pro-tumor phenotype, and were proportionally decreased in response to ACTM-838 treatment (except OXPHOS TAMs) [51–53] (Figure 7B, 7C). SPP1-C1Qa/b and proliferating TAM subsets showed a significant increase in expression of MHCI/II, CD74, CLEC2D and a significant decrease in C1Qa/b/c and SPP1 expression with ACTM-838 treatment (Figure 7D, 7E). Notably, CD74 is a M1-like macrophage marker associated with good prognosis and functions as a chaperone for MHCII transport [54]. CLEC2D, a DAMP receptor, augments histone-CpG DNA responses in macrophages [55]. Nos2 glycolytic and OXPHOS TAMs showed a significant decrease in the immunosuppressive SPP1 expression profile (Supplementary Figure 11A, 11B). Collectively, these data suggest the immunosuppressive TAM subsets are reprogrammed towards an anti-tumor antigen-presenting immune phenotype.

Anti-tumor TAM subsets (CD74 and CD163-CD206 phagocytic TAMs) exhibited high expression of MHCII and CSF3R, and a 3- and 7-fold increase with ACTM-838 treatment, respectively (Figure 7B, 7C). CD206^+^ TAMs can produce CXCL9, are associated with antigen presentation and regulate anti-tumor cDC1-CXCR3^+^ lymphocyte assembly in the TME [38, 56] (Supplementary Figure 11D). CD74 TAMs exhibited a further increase in MHCII and CD74, and a decrease in immunosuppressive C1Qa/b and AXL genes (Supplementary Figure 11C).

Within the tumor-associated neutrophil population, neutrophils expressing glycolytic and degranulation markers showed a 2–3-fold decrease with ACTM-838 treatment (Figure 7B). Interestingly, glycolytic neutrophils showed a significant increase in activation markers (e.g., PDL1, MHCI and II, Nos2) and decrease in immunosuppressive markers (e.g., CD44) with ACTM-838 treatment (Figure 7F) suggesting activation towards neutrophil-derived cross-presenting DC function. Similarly, degranulation neutrophils showed a significant increase in MHCI expression and downregulation of IL-1β (Supplementary Figure 11E). IL-1β is a key driver of immunosuppressive neutrophils, whereas MHCI positive neutrophils are associated with antigen cross-presentation and function like DCs [57]. Overall, ACTM-838 induced activation of myeloid subsets, antigen cross-presentation and priming of T-cells leading to cytolytic T-cell activity in the TME.

## DISCUSSION

While ICB and other T-cell focused immunotherapies represent significant advances in the treatment of cancer, there remains a high unmet need for immunotherapies that can make the TME permissive to anti-tumor immunity. Multiple clinical trials have underscored the significance of myeloid-mediated resistance mechanisms to ICB. TAMs, MDSCs, neutrophils and DCs are major tumor-infiltrating myeloid cells. Optimal anti-tumor immunity needs to engage both the innate and adaptive immune system and overcoming myeloid resistance mechanisms is key. Myeloid-targeted therapies in cancer have been tested in clinical trials, however the complexity and plasticity of myeloid subsets have challenged clinical efficacy [58]. Type I IFN-inducing agents show promise in shifting the state of myeloid resistance, but the cytokine levels needed to activate the TME cannot be tolerated systemically [59]. Therefore, there is an unmet need to deliver therapeutic payloads to the TME of primary and metastatic lesions via systemic administration.

The parental *S.* Typhimurium VNP20009 was evaluated in the clinic and demonstrated low levels of tumor colonization at the highest doses tested, though with DLTs that limited higher dosing due to persistent bacteremia and cytokine storms [29]. We hypothesized that if the bacterial strain could be engineered to have reduced systemic toxicity, higher doses and ensuing tumor colonization could be achieved. We further hypothesized that using the bacterial vector for gene delivery of immune-modulatory payloads to specific cell types in the TME could enhance anti-tumor efficacy. Thus, STACT was created from the parental VNP20009 strain through genome engineering to attenuate TLR-mediated production of systemic proinflammatory cytokines and eliminate bacterial features that cause immunosuppression of CD8^+^ T-cell responses, enabling higher dosing in the clinic to achieve tumor colonization. STACT has been precision genome edited to be a live, programmable and tolerable systemically delivered immunotherapy, where genes regulating expression or characteristics of LPS, flagella, curli fimbriae and asparaginase-II were deleted, resulting in a less pathogenic microbe. STACT can also harbor plasmids that deliver DNA-encoded therapeutic payloads for improved efficacy. In addition to the eukaryotic expression cassette, the STACT chassis provides payload plasmid maintenance without antibiotic-resistance cassettes. Thus, STACT is sensitive to frontline antibiotics, providing an off-switch in the clinic if needed. Consistent with our hypotheses, ACTM-838, the STACT chassis carrying a plasmid that encodes IL-15/IL-15Rα and a constitutively active form of STING, exhibited a significantly decreased acute systemic proinflammatory cytokine response compared to VNP20009 in tumor-bearing mice upon IV dosing.

Having established the potential for an increased therapeutic index over VNP20009, we tested ACTM-838 in syngeneic and spontaneously metastasizing murine tumor models. ACTM-838 exhibited potent dose-dependent anti-tumor monotherapy efficacy across multiple mouse tumor models, showing its utility in primary and metastatic settings. Importantly, the lack of tumor growth upon rechallenge of cured animals suggested durable anti-tumor immunological memory mediated by CD8^+^ T-cells. Consistent with the hypothesized mechanism of ACTM-838, myeloid cells treated with ACTM-838 showed high expression of PDL1, correlating with activation status and antigen presentation. Of note, combinations of anti-PD1 with either STING agonists or IL-15 have shown improved efficacy in the clinic [60, 61]. Combination treatment of anti-PD1 with ACTM-838 showed durable synergistic anti-tumor efficacy and cures in immune checkpoint blockade refractory and sensitive settings, with immune excluded or inflamed microenvironments, respectively. The upregulated expression of myeloid PDL1 upon ACTM-838 treatment likely opens possibilities in immune checkpoint refractory settings in the clinic, independent of baseline PDL1 expression.

ACTM-838 requires high local concentrations of purines (e.g., adenosine) to enrich and proliferate extracellularly, and tumor-resident myeloid cells for intracellular uptake. Primary and metastatic tumors, as well as tumors of patients relapsed or refractory to immune checkpoint blockade therapies, exhibit high levels of adenosine and immunosuppressive myeloid cells in the TME, providing fertile ground for ACTM-838 to proliferate and deliver its payloads. Enzymes CD73 and CD39 convert ATP to AMP and adenosine and are upregulated in both tumor and immune cells mediating immunosuppression. Blocking CD73 and CD39 to reduce immunosuppression in the TME is an ongoing area of interest in the clinic [43]. Here, though, we are exploiting this property of tumors to deliver genetic payloads that shift the TME to an anti-tumor immune-permissive state.

Once TME colonization is established, ACTM-838 acts across the cancer-immunity cycle [62]. ACTM-838 uptake by phagocytosis triggers myeloid cell activation via the STACT chassis, itself, activating the cGAS-STING pathway. This pathway bridges innate and adaptive immunity by promoting cross-priming, antigen presentation and activation of T-cells via type I IFN dependent and independent mechanisms. STING payload expression further augments this activity, noting that it is advantageous to activate the STING pathway within APCs [15]. Consistent with the pathway modulation and changes in immune contexture we observed, the hypothesized mechanism downstream of payload expression is based on the established activity of type I IFNs and IL-15. The STING-induced type I IFNs also activate NK-cells, stimulating them to attack tumor cells, thus releasing tumor antigens for myeloid presentation and enabling the priming of a new T-cell response in a now immune-permissive environment [14]. IL-15 activates cytolytic effector T-cells and NK-cells without increasing immunosuppressive Tregs, thus providing synergistic efficacy in activating both the innate and adaptive immune cells [14, 63]. Consistent with this mechanism, ACTM-838, as a single IV dose, induced significant and comprehensive activation of the immunosuppressive TME, targeting both innate and adaptive immune cell subsets. Myeloid activation with increased MHCI/II antigen presentation and expression of co-stimulatory molecules CD80/86 with maintained phagocytic capacity (CD206/CD163) was observed in ACTM-838 treated myeloid cells. Antigen-presenting DC subsets (Flt3^+^ pDCs, Batf3^+^ cDC1) with high MHCII and antigen cross-presenting mature DCs with high MHCI and II were increased with ACTM-838 treatment. Immunosuppressive neutrophil subsets showed an increase in MHCII and MHCI (H2-K1/Q6) suggesting cross-presentation [64, 65]. ACTM-838 also increased infiltration of T-, NK- and B-cells and cytolytic activity of T-cells, as well as a reduction in CD4^+^ Tregs and exhausted CD8^+^ T-cells. An increase in B-cells, T-cells and CXCL13 suggests potential tertiary lymphoid structure formation with ACTM-838 treatment [66], which remains to be studied. Thus, the ACTM-838 STACT chassis and IL-15plex-eSTING payload activities impact multiple mechanisms in the cancer immunity cycle.

ACTM-838’s activity in modulating the TME toward an immune-permissive phenotype has potential beyond efficacy as a monotherapy or in combination with anti-PD1, as was the focus of the work presented here. We hypothesize that combination with ACTM-838 could potentiate the activity of immunotherapies whose activity might be hindered by an immune-suppressive TME. For example, one mechanism inhibiting the full potential of CAR-T [67, 68] or T cell engager (TCE) [69] therapies is the immune-suppressive microenvironment of many solid tumors. Additionally, it should be noted that the STACT platform allows for the expression of other payloads beyond the eSTING and IL-15-plex payloads delivered by ACTM-838, suggesting that the platform enables numerous immune modulatory possibilities. ACTM-838 is currently being assessed for safety in a phase I clinical trial (NCT06336148). Successful completion of this study will bode well for ACTM-838 as a monotherapy and in combination therapy, as well as for additional applications of the STACT platform itself.

In conclusion, ACTM-838 is a live attenuated bacterial immunotherapy for which the following has been demonstrated in preclinical studies – (1) enhanced safety and tolerability after IV dosing in animal models via reduced systemic cytokines; (2) tumor-specific enrichment over healthy tissues; (3) cell-type specific uptake and payload delivery only to phagocytic APCs, not epithelial or endothelial cells; (4) attenuated bacterial immunosuppressive pathways to promote CD8^+^ T-cell activation in the TME; (5) ability to carry DNA plasmids encoding multiplexed immunostimulatory payloads capable of activating durable anti-tumor innate and adaptive immunity. Localized delivery of eSTING and IL-15/IL-15Rα via ACTM-838 exhibits effective synergistic activity to engage both innate and adaptive immunity in the tumor and generate a durable T-cell mediated systemic anti-tumor immune response.

## MATERIALS AND METHODS

### ACTM-838 tissue biodistribution *in vivo*

1e5 EMT6 TNBC cells were implanted into the mammary fat pad of 6–8-week-old female BALB/c mice (Charles River Labs). Once tumors reached an average tumor volume of 50–100 mm^3^, animals were dosed IV with ACTM-838 (3.8e7 CFU/animal). Mice were euthanized at the scheduled times with CO_2_ exposure and exsanguination via cardiac puncture for whole blood collection. Tissues were harvested (5 mice per timepoint per dose) at 2 hr, 6 hr, and at days 1, 2, 4, 7, 14, and 21 post-treatment. Details on tissue processing and colony counting are in Supplementary Methods.

### *In vivo* cellular internalization and cytokines assessment

1e5 EMT6 tumor cells were implanted into 6-8-week-old female BALB/c mice (Charles River). When tumors reached 50–100 mm^3^, 3e7 CFU (VNP20009/ACTM-838)/mouse was administered IV. At days 1 or 7 post-ACTM-838 treatment, whole blood and tissues were collected on ice and tissues were dissociated with collagenase using a mouse tumor dissociation kit (Miltenyi Biotec, 130-096-730). Details on flow cytometry analysis and cytokine assessments are in Supplementary Methods.

### Cellular internalization and activation in primary human cells

Human MDM (M0- and M2-like, Supplementary Methods) were treated with ACTM-838 (MOI 40). 50 μg/mL of gentamicin was added after 1 hour of treatment to control the growth of extracellular bacteria. Supernatant of MDMs were collected for multiplex cytokine detection using Legendplex (BioLegend, 740365, 741270) at 48 hours, and myeloid activation was analyzed by flow cytometry (Supplementary Methods).

### Mouse efficacy studies

6-8-week-old female BALB/c or C57Bl/6 mice were implanted in the mammary fat pad with 1e5 EMT6 tumor cells or in the flank with 5e5 MC38 colon tumor cells respectively. Once tumors grew to an average of 50–100 mm^3^, mice were randomized by tumor volume and dosed intravenously via the tail vein with either PBS or increasing doses of ACTM-838 (1e7, 3e7 or 6e7 CFU/mouse) as monotherapy. For anti-PD1 combination studies, mice were dosed with ACTM-838 (3e7 CFU/mouse) and/or anti-PD-1 antibody (100ug, BioXcell, CP151). ACTM-838 was given as a single dose on day 0 intravenously, while anti-PD-1 antibody was dosed intraperitoneally every 3–4 days starting on Day -2. Tumors were measured using electronic calipers 2x/week for efficacy and animals were evaluated for tolerability. Tumor rechallenge and metastasis studies are described in Supplementary Methods. All animal procedures were conducted in accordance with the guidelines established by the Institutional Animal Care and Use Committee (EB17-010-128).

### *In vivo* study design and sample collection for mechanism of action studies

Treatment regimens and study design is as described above. For bulk RNAseq, EMT6 tumors 4 days post-treatment were snap frozen in liquid nitrogen. This was followed by RNA extraction and standard Illumina mouse polyA library preparation. Data preprocessing and analysis are in Supplementary Methods.

For single cell RNAseq, EMT6 tumors post-treatment were dissociated on ice into the single cell suspension using the Miltenyi Biotec mouse tumor dissociation kit (130-096-730), followed by EasySep dead cell removal kit (StemCell, 17899) according to manufacturer’s protocol. The 10X Single Cell Fixed RNA Mouse Transcriptome Probe Kit (PN-1000491) and 10X Single Cell Fixed RNA Hybridization and Library Kit (PN-1000415) was used to prepare the libraries, with Chromium X to prepare GEMs. Study design, data preprocessing and analysis are in Supplementary Methods.

For flow cytometry analysis, EMT6 tumors were implanted and treated as described above with 6e7 CFU/mouse and dissociated into single cell suspension using the Mouse Tumor Dissociation Kit (Miltenyi Biotec, 130-096-730). Cells were stained with live-dead dye (Thermo, L34976) followed by Fc Block (Biolegend, 156604), and pre-conjugated antibodies (see Supplementary Methods) were incubated with samples for 30mins. For intracellular markers, cells were permeabilized (Thermo, 00-5523-00) prior to staining and flow analysis. Data preprocessing, antibody panels and analysis are in Supplementary Methods.

## SUPPLEMENTARY MATERIALS



## Abbreviations

APC: antigen presenting cells
CCL: C-C motif chemokine ligand
CD: cluster of differentiation
CFSE: carboxyfluorescein succinimide ester
CTT: C-terminal tail
CXCL: CXC motif chemokine ligand
DC: Dendritic cells
HMECs: human mammary epithelial cells
HUVEC: human vascular endothelial cells
IFN: interferon
IL: interleukin
IRF: interferon regulatory factor
LOD: limit of detection
MDM: monocyte derived macrophages
MHC: major histocompatibility complex
moDCs: monocyte-derived DCs
MOI: multiplicity of infection
ND: not detected
OXPHOS: oxidative phosphorylation
PBS: phosphate buffered saline
PDL1: Programmed death-ligand 1
pg/ml: picogram/milliliter
RNAseq: ribonucleic acid sequencing
scRNAseq: single-cell ribonucleic acid sequencing
TAM: tumor-associated macrophages
TCRβ: T-cell receptor beta
TME: tumor microenvironment
TNF: tumor necrosis factors
