# Supplementary material for: ACTM-838, a novel systemically delivered bacterial immunotherapy that enriches in solid tumors and delivers IL-15/IL-15Rα and STING payloads to engage innate and adaptive immunity in the TME and enable a durable anti-tumor immune response
Source: Oncotarget. 2025 Oct 6;16:721–40. doi: 10.18632/oncotarget.28769 (PMC12758031; doi:10.18632/oncotarget.28769)
Supplement: Supplementary file 1 [file oncotarget-16-28769-s001.pdf]

ACTM-838, a novel systemically delivered bacterial immunotherapy that enriches in solid tumors and delivers IL-15/IL-15Rα and STING payloads to engage innate and adaptive immunity in the TME and enable a durable anti-tumor immune response

SUPPLEMENTARY MATERIALS

SUPPLEMENTARY DATA

Enhanced safety and sustained tumor colonization of ACTM-838, A STACT carrying plasmid encoding IL-15plex and eSTING payloads

The STACT platform was developed using the parental *S.* Typhimurium VNP20009 strain via strain engineering to eliminate bacterial mediated T-cell immunosuppressive factors and reduce Toll-like receptor (TLR)-mediated systemic cytokine responses associated with dose-limiting toxicities (DLTs) in subjects treated with VNP20009 [1]. The STACT strain was engineered with deletions in genes encoding the flagellum, an external structure that enables invasion of epithelial and endothelial cells, allows for Salmonella immune escape, and acts as a TLR5 agonist [2]. Electron microscopy confirmed that the flagellin gene deletions in STACT led to loss of the flagellum (Supplementary Figure 1A). This modification prevented STACT (ACTM-838) internalization into primary human epithelial (HMECs) or endothelial (HUVEC) cells but not M2 macrophages, a cell type capable of active phagocytosis (Supplementary Figure 1B). In contrast, VNP20009 did not exhibit this cell-type specificity for internalization. Despite being readily internalized by M2 macrophages, STACT is subsequently eliminated, potentially allowing for plasmid delivery as described previously [3] (Supplementary Figure 1C). Additionally, deletion of genes involved in the curli fimbriae in STACT prevented the formation of biofilms indicated by the absence of the red, dry, and rough (rdar) morphotype compared to VNP20009 (Supplementary Figure 1D). Biofilms enable salmonella to escape immune and antibiotic mechanisms and persist *in vivo* [4].

VNP20009 exhibits purine auxotrophy and has been previously shown to colonize syngeneic mouse tumors and patient tumors at high IV doses [5]. Like VNP20009, STACT is also a purine auxotroph and is unable to grow without purine/adenine supplementation (Supplementary Figure 1E), allowing for tumor-specific enrichment in the purine-rich (e.g. adenosine, ATP) TME [6].

Humans exhibit differential TLR4-mediated IL-6 responses depending on the LPS acylation status where tetra- and penta-acylated LPS result in dampened TLR4 cytokine responses in comparison to hexa-acylated LPS [7]. To attenuate a systemic IL-6 response, the STACT genome was further modified to alter the LPS acylation state to the penta-acylated state, compared to VNP20009 and WT, which each express both penta- and hexa-acylated LPS, as shown previously [8, 9] (Supplementary Figure 1F).

In contrast to VNP20009, STACT was designed to carry plasmids that encode distinct payloads (RNA, peptides, proteins and gene-editing effectors) for improved therapeutic efficacy. ACTM-838 comprises of a STACT chassis strain encoding plasmids with 2 engineered immune-stimulatory payloads, human IL-15 bound to IL-15Ra (hIL-15plex) and a constitutively active variant of STING (eSTING) (Supplementary Figure 1G). Both payloads are active across species in human and murine systems (data not shown). *asd*$^{+}$ vector/Δ*asd* complementary system was used for plasmid maintenance, where the *asd* gene was deleted from the bacterial genome and inserted into the plasmid. This system allows for plasmid maintenance without the use of antibiotics as the bacteria requires the *asd*-containing plasmid to survive in the absence of diaminopimelic acid (DAP) [10]. This plasmid is maintained in ACTM-838 *in vitro* and *in vivo* without the need for antibiotics, allowing for multiple copies of plasmid per bacterium (Supplementary Figure 1H; data not shown). Strain identity was verified by PCR, confirming alterations to the genomic plasmid and payload plasmid sequence was verified by Sanger sequencing.

Salmonella secretes L-asparaginase, which inhibits the T-cell response by depleting local L-asparagine concentration in the TME [11]. STACT has an engineered asparaginase gene deletion, lacking the ability to convert extracellular L-asparaginase to aspartic acid (Supplementary Figure 1I). As a result, T-cells treated with prototype STACT strains exhibit restored TCRβ expression and T-cell cytokine secretion comparable to media control (Supplementary Figure 1J).

Additional genomic modifications to STACT were aimed at reducing *Salmonella*-related toxicity and improving its safety profile to enable higher doses in the clinic. We assessed cytokine responses on days 2 and 7 after a single IV dose of either VNP20009 or ACTM-838

in EMT6 triple negative breast cancer (TNBC) orthotopic tumor-bearing mice. The T-cell cytokine IL-2 was very low or below LOD (<10 pg/ml) in most conditions (Supplementary Figure 2). Compared to VNP20009, treatment with ACTM-838 resulted in significantly less cytokine levels activated by TLR2 (IL-1β, IL-10), TLR4/9 (IL-6, IL-10, TNFβ), and TLR9 (IL-6, TNFα) as well as chemokines CXCL1, CXCL10 and CCL2, suggesting that the genomic modifications in STACT dampen the proinflammatory cytokine response in the periphery (Figure 1A; Supplementary Figure 2).

Tolerability of ACTM-838 and VNP20009 was compared by administering a single IV bolus of increasing doses in female C57BL/6 mice. No mortalities were observed at 1×10^8 or 2×10^8 CFU/animal of ACTM-838. All mice administered with 2×10^8 CFU/animal ACTM-838 exhibited piloerection and lethargy 10 days post-dosing. In addition, mice lost 3–20% of their starting body weight by day 10, but recovery was noted by day 19 post-dose. However, mice treated with VNP20009 exhibited 100% mortality at 1×10^7 CFU and 2×10^7 CFU/animal, consistent with previous literature [12]. Thus, ACTM-838 was at least 50-fold better tolerated than VNP20009 in this model.

To confirm tumor colonization and understand biodistribution kinetics, EMT6 orthotopic tumor-bearing mice were dosed intravenously with a single dose of ACTM-838. Organs and tissues were collected, processed, and live CFU plating was performed at various timepoints to measure colonization. ACTM-838 was not detected in the urine or feces of EMT6 tumor-bearing BALB/c mice at any timepoint and ACTM-838 was rapidly cleared from blood within 4 days of IV dosing. Rapid biodistribution of ACTM-838 was observed in all tissues and enrichment in tumor was observed within 24 hours. Other organs showed rapid decline of ACTM-838 colonies with time while tumors exhibited persistent colonization over time (Figure 1B; Supplementary Figure 3; Supplementary Table 3). Despite the attenuating genomic modifications, ACTM-838 continued to show enriched tumor colonization at 1000-fold higher levels at day 21 as compared to spleen and liver, which are the natural sites for *Salmonella* clearance, and colonization was either not detectable or detectable at just above limit of detection in all other tested tissues (LOD <20 CFU) (Figure 1C; Supplementary Table 3).

Plasmids delivered by ACTM-838 enable functional secreted IL15plex and induces eSTING mediated IFNβ activity in human target cells

While genomic modifications on the chassis reduce virulence and systemic inflammation, ACTM-838 carries multiple copies of plasmid ADN-838 comprising of 2 genetically encoded payloads for delivery in the tumor milieu: (1) IL-15plex- a complex of IL-15 bound to IL-15Rα sushi domain i.e., IL-15 superagonist [13], and (2) eSTING- an engineered variant of human STING with 2 mutations, rendering it constitutively active, as well as a replacement of the C-terminal tail (CTT) with the Tasmanian devil STING CTT to increase type I IFN activity and reduce NF-κB mediated IL-6 secretion [14–16] (Supplementary Figure 1G, 1H).

To evaluate the ability of the plasmid delivered by ACTM-838 to enable payload expression in target mammalian cells, HEK293 STING KO IFN-reporter cells were transfected with plasmids expressing 1 payload (IL-15plex or eSTING), IL-15Rα sushi domain only and eSTING with deleted CTT (nonfunctional payload control), or IL-15plex + eSTING. ACTM-838 plasmid induced high IL-15plex protein levels similar to IL-15plex alone (Supplementary Figure 3A). No IL-15plex was detected in plasmids containing eSTING alone or inactive payloads. ACTM-838 plasmid also induced IFNβ reporter activity suggestive of eSTING payload expression although reporter activity was slightly lower in dual payload plasmid than control plasmids containing eSTING alone (Supplementary Figure 3B). Together, these data indicate that ACTM-838 plasmid encodes functional IL-15plex and eSTING payloads.

To assess the ability of ACTM-838 to deliver payloads via cellular internalization, ACTM-838 was transfected into HEK293 cells using a transfection reagent to increase internalization efficiency, as ACTM-838 cannot infect epithelial cells due to the genomic deletion of flagella (Supplementary Figure 1B). A dose-dependent increase of IL-15plex secretion and eSTING mediated IFN-β reporter activity was observed with ACTM-838 (Supplementary Figure 4C, 4D). Cells directly transfected with plasmid also secreted IL-15plex, but no IL-15 was detected in negative control plasmids containing inactive IL-15 sushi domain and eSTING with deleted CTT as payloads. Furthermore, no IL-15 was detected in the supernatant of bacteria incubated in media without mammalian cells (Supplementary Figure 4E). These data suggest that ACTM-838 STACT chassis can effectively deliver its plasmids once internalized and enable payload expression and activity in target mammalian cells.

To test mechanism of uptake and payload expression in human macrophages, we used human THP-1-Dual macrophages, a model cell line measuring STING activity via IRF-driven luciferase secretion. THP-1 macrophages were treated with increasing MOI of pHrodo dye labeled ACTM-838 for 1 hour. Phagosomal internalization was measured post-treatment where pHrodo-labeled ACTM-838 fluoresce in acidic environments (e.g., phagolysosomes), which can be readily measured when present in THP-1-Dual cells. ACTM-838 uptake levels were observed to be dose dependent in M0-, M1- and M2-like THP-1-Dual macrophages (Supplementary Figure 4F). After 48 hours, ACTM-838 treated macrophages

showed a dose dependent increase in eSTING mediated IRF luciferase reporter activity which correlated with lysosomal uptake (Supplementary Figure 4G). To further confirm the phagocytic ACTM-838 uptake in human myeloid cells, healthy donor PBMCs were treated with pHRodo-labeled ACTM-838, which also showed similar results where only cells capable of active phagocytosis such as monocytes, DCs and B-cells exhibited ACTM-838 uptake, while other cell types such as T-cells did not (Supplementary Figure 5).

SUPPLEMENTARY METHODS

Electron microscopy for flagella visualization

Exponential phase bacteria (VNP20009 (ATCC #YS1646) and STACT) were fixed in 2.5% glutaraldehyde in 0.1M phosphate buffer. Bacteria were imaged using the FEI Tecnai 12 Transmission Electron Microscope electron microscope at 60X magnification.

Congo red rdar phenotype assay

Congo Red dye binds to amyloid proteins such as curli fimbriae to create a Red Dry And Rough (RDAR) phenotype. Congo red plates were prepared by mixing soytone (10 g/L), yeast extract (5 g/L), Congo red (40 mg/L), and Coomassie brilliant blue G-250 (20 mg/L) in water and then brought up to 1L volume and autoclaved on wet cycle. After cooling, 30–35 mL was dispensed into plastic plates under sterile conditions. After plates solidified, they were transferred to 4ºC for storage. 2 mL of liquid culture media was inoculated with strains of interest and grown at 37ºC overnight to stationary phase. 5 µL was spot plated Congo red plates incubated at 37ºC for 16 hours. The plate was then transferred to a 30ºC incubator for incubation. Plates were imaged daily for up to 7 days to observe bacterial colony morphology.

LPS characterization

Bacterial pellets of VNP20009 or ACTM-838 were suspended in a PBS/chloroform/methanol solution. After a 10-minute incubation, glacial acetic acid was added to the suspension and any remaining cell pellets were disrupted by sonication. The resulting suspensions were heated at 90ºC for 90 min to hydrolyze the LPS into lipid fragments before cooling to ambient temperature. Chloroform was added to each sample and the contents were vortexed and then centrifuged. The organic (lower) layer was transferred to a fresh centrifuge tube and mixed with purified water. The samples were vortexed again and then centrifuged. The organic layer (lower) was again transferred to a new centrifuge tube before drying under nitrogen. The samples were then reconstituted in a 4:1 chloroform:methanol solution and mass spectrometry analysis was performed (MS-QTOF (ESI)).

Asparginase activity

T cells isolated from 2 BALB/c spleens and overnight bacterial cultures were added to RPMI media containing FBS. Supernatant was harvested after 24 hours for CBA and cells were stained for TCRβ. The asparaginase activity assay was performed in accordance with the manufacturer's instructions (BioVision, K754-100). VNP20009 and ACTM-838 were centrifuged at 10,000 rpm for 2 minutes, the cell pellets were homogenized on ice with 100 µL of ice-cold asparaginase assay buffer by vortexing and centrifuging at 13,500 rpm for 10 minutes to remove insoluble materials. 40–48µL of asparaginase assay buffer was added to a clear, flat-bottom 96-well plate and volume was adjusted in each sample well to 50µL with the supernatant. Positive control from the provided kit was used as directed. Plate was read on a plate reader for colorimetric assay.

Purine auxotrophy

Overnight stationary phase cultures of WT *S.* Typhimurium (14028S, ATCC), VNP20009 (ATCC, YS1646), and ACTM-838 were grown in 2X M9 minimal media with glucose at 37ºC, 225 rpm. Control groups were supplemented with 40 µL of water and adenine groups were supplemented with 40 µL 10mM adenine. Bacterial growth was monitored for approximately 19 hours in a spectrophotometer shaking at 37ºC with OD600 readings taken every 15 minutes.

Plasmid copy number quantification

Plasmid copy number assay (PCN) was performed on 1e9 CFU from thawed injection stocks of ACTM-838 and VNP20009, followed by centrifugation at 17,000g for 2 minutes at room temperature. Cell pellets were resuspended in 100ul nuclease-free water. Samples were heated in heat block for 10 mins at 95 +/− 5 degrees. Sample was placed on dry ice to freeze for 5 minutes. This heating and cooling process was repeated 2 times, followed by centrifugation at 17,000 g for 5mins at room temperature. DNA concentration in supernatant was measured by Nanodrop at 260/280 nm. iQ multiplex powermix (Biorad, 172-5849) was used per manufacturer's instructions for qPCR and run on the CFX96 Real-Time PCR Detection System for DNA quantification. Plasmid copy number was determined by qPCR analysis of the genes for asd (found on plasmid) and invA, an essential gene found in Salmonella.

Payload detection in mammalian cells

Human THP-1-Dual cells (Invivogen, thpd-nfis) stably express two inducible secreted reporter genes, Lucia luciferase regulated by IRF pathway or STING activity, and SEAP regulated by NF-kB pathway. THP-1-Dual cells were cultured in RPMI media with 2 mM

L-glutamine, 25 mM HEPES, 10% heat inactivated FBS, 100 ug/ml Normocin and penicillin-streptomycin (100 U/ml – 100 ug/ml) per manufacturer instructions. Cells were differentiated into M0-like macrophages using 20 ng/ml PMA overnight, followed by washout and incubation with fresh RPMI media for 3 days. M1-like or M2-like macrophages were differentiated from M0-like macrophages using IFN (25 ng/ml) + LPS (250 ng/ml) or IL-4 + IL-10 (20 ng/ml each) respectively for 48 hours at 37 degrees. Human THP-1-Dual macrophages labeled with pHRodo dye were treated with increasing MOI (1, 5, 20, 40) of ACTM-838 for 1 hour, followed by gentamycin treatment to kill extracellular bacteria. After 48 hours, supernatant was collected, and STING-mediated IRF-Lucia reporter activity was measured by measuring luciferase Relative Light Units (RLU) using a plate reader. cGAMP stimulation was used as a positive control.

Payload detection in HEK293 reporter and HEK293T cells

HEK293 (ISRE SEAP) Dual-Null cells (Invivogen, hkb-isgkostg) were transfected with each plasmid. HEK293 Dual-Null and HEK293T cells (ATCC, CRL-3216) were grown according to manufacturer's instructions. After 48 hours, supernatant was collected to measure eSTING-mediated IFNβ luciferase reporter activity via plate reader. Expression of human IL-15plex was measured via ELISA (PBL, 41702) 48 hours post-transfection. CMV promoter-driven GFP was used was used as a transfection efficiency control for each plasmid and used to normalize the payload expression. As ACTM-838 is not internalized by non-phagocytic HEK293 cells, ProJect (Pierce, 89850P) was used to promote internalization. ACTM-838 was incubated for 1 hour, followed by gentamycin treatment to remove the non-internalized bacteria. After 48 hours, supernatant was collected to measure eSTING-mediated IFNβ luciferase reporter activity using Quanti-Luc 4 (Invivogen, rep-qlc4lg1) via plate reader. Expression of human IL-15plex was measured via ELISA 48 hours post-transfection.

Murine efficacy studies

EMT6 (ATCC, CRL-2755) were grown in Waymouth's MB medium with 2mM glutamine per supplier instructions. MC38 cells (Sigma, SCC172) were grown in DMEM high glucose medium with 10% FBS per supplier instructions. 6–8-week-old female BALB/c or C57Bl/6 mice were implanted in the mammary fat pad with 1e5 EMT6 tumor cells or in the flank with 5e5 MC38 colon tumor cells respectively ($N = 10$ per treatment arm). Once tumors grew to an average 50–100 mm^3, mice were randomized by tumor volume and dosed IV via the tail vein with either PBS or increasing doses of ACTM-838 (1e7, 3e7 or 6e7 CFU/mouse) as monotherapy. For anti-PD-1 combination studies, ACTM-838 (3e7 CFU/mouse) and/or anti-PD-1 antibody (100ug, BioXcell, CP151) were administered as a monotherapy or in combination. ACTM-838 was given as a single dose on day 0 via the tail vein (IV), while anti-PD-1 antibody was dosed intraperitoneally every 3 to 4 days starting on day -2. Tumors were measured using electronic calipers 2x/week for efficacy and animals were evaluated for tolerability. Cured animals were evaluated for tumor regrowth for 30 days before tumor rechallenge. For tumor rechallenge studies, 1e5 EMT6 or 5e5 MC38 fresh tumor cells were injected into the contralateral mammary fat pad or flank respectively of cured mice after 30–35 days in remission post-ACTM-838 treatment. Rechallenged tumors were measured 2x/week for 30 days.

To evaluate the durable memory response after ACTM-838 treatment, each cured animal received IP injections of either 100 mg CD8β T-cell depleting antibody (BioXcell, BE0223) or an IgG isotype control antibody 3 days prior to tumor rechallenge with 1e6 EMT6 cells implanted orthotopically into the opposite mammary fat pad (day 56 post-initial tumor implantation). Blood samples were analyzed to confirm CD8+ T-cell depletion by flow cytometry. Average circulating CD8+ T-cells were 5.72% of total white blood cells for mice treated with the isotype control, and 0.48% for mice treated with anti-CD8β antibody.

EMT6 lung metastasis model: 6–8-week-old naïve female BALB/c mice were implanted with 1e5 EMT6 tumor cells via the tail vein. Three days after tumor cells were implanted, mice were dosed with a fixed volume of 200 µL intravenously (tail vein) with either PBS (control), or ACTM-838 at 6e7 CFU/mouse. Tumors were allowed to grow until day 11 post-dose (day 14 post-tumor implantation) and mice were sacrificed. Lungs were collected and stained with Indian dye ink to count metastatic nodes.

MMTV-PyMT GEMM Model: 6–8-week-old MMTV-PyMT GEMM female mice (Jackson Laboratory) showing 1–2 spontaneous palpable tumors in the mammary fat pads were administered 6e7 CFU of ACTM-838/mouse intravenously. Tumor volumes of mammary fat pads # 2 and 7 were measured over time in the PBS control and ACTM-838-treated mice. On day 36, the cumulative tumor volume across all 10 tumors of each mouse were measured as well as the total number of tumors per animal. At endpoint (day 45), lungs were dyed with India ink to visualize the metastatic nodules and counted by eye.

Cellular internalization and activation in primary human cells

Human monocyte-derived macrophages (MDMs) were derived from human CD14+ monocytes (Lonza) in Immunocult human MDM medium (Stemcell, 10961). Human mammary epithelial cells (HMEC) were cultured in mammary epithelial cell basal medium (ATCC, PCS-600-030) with mammary epithelial cell growth kit (ATCC, PCS-600-040) per manufacturer's instructions. Primary human umbilical vein endothelial cells (HUVEC)

(ATCC, PCS-100-010) were cultured in F-12K base medium (ATCC, 30-2004), heparin (Sigma, H3393), FBS (Gibco, 220623) and endothelial growth supplement ECGS (Corning, CB-40006). Human MDMs, HMEC and HUVEC were plated in 24-well cell culture dishes with 5e5 cells per well in 500 µL RPMI media (Gibco). Cells were treated with MOI 50 of either VNP20009 or ACTM-838 at a 100 µL volume. Treated cells were allowed to incubate for 1 hour at 37°C, washed, then cell culture media containing 100 µg/mL gentamicin was added. Cells were then allowed to incubate for an additional hour, washed, then lysed with Triton-X 100 to release intracellular bacteria. Bacteria were then plated on LB agar plates, incubated at 37°C, and quantified the following day by colony counting.

For assessment of lysosomal uptake, pHRodo dye-labeled (Thermo, P35357) VNP20009 or ACTM-838 were used for treatment of MDMs, HUVECs and HMECs. Treated cells were allowed to incubate for 1 hour at 37°C, washed, then cell culture media containing 100 µg/mL gentamicin was added. Primary human cells were then assessed for pHrodo positivity by flow cytometry.

To assess degradation of internalized bacteria, primary human M2 macrophages were incubated with ACTM-838 at an MOI 75 for 1 hour which was followed by gentamycin treatment. Macrophages were then treated for 1 hour with chloroquine (200 uM) and washed with PBS to enable detection of internalized bacteria. Internalization was measured at each timepoint by lysing the cells with Triton-X and CFU plating.

Bulk RNAseq analysis

EMT6 tumors on day 4 post-treatment were snap frozen in liquid nitrogen, followed by RNA extraction and standard Illumina mouse polyA library preparation. Sequence reads were trimmed to remove possible adapter sequences and nucleotides with poor quality using Trimmomatic v.0.36. The trimmed reads were mapped to the Mus musculus GRCm38 reference genome available on ENSEMBL using the STAR aligner v.2.5.2b. Unique gene hit counts were calculated by using featureCounts from the Subread package v.1.5.2. After extraction of gene hit counts, the gene hit counts table was used for downstream differential expression analysis. Using DESeq2, a comparison of gene expression between PBS and ACTM-838 samples was performed. The Wald test was used to generate *p*-values and log2 fold changes. Genes with an adjusted p-value < 0.05 and absolute log2 fold change >1 were considered differentially expressed genes. Differential pathway enrichment analysis was performed using the multiGSEA R package. Boxplots were generated using the ggplot2 R package.

Single-cell RNAseq and data preprocessing

EMT6 tumors were dissociated on ice into the single cell suspension using the Miltenyi Biotec mouse tumor dissociation kit (130-096-730), followed by EasySep dead cell removal kit (StemCell, 17899) according to manufacturer’s protocol. 10X Single Cell Fixed RNA Mouse Transcriptome Probe Kit (PN-1000491) and 10X Single Cell Fixed RNA Hybridization & Library Kit (PN-1000415) were used to prepare the libraries and Chromium X to prepare GEMs.

Raw sequencing reads were aligned to the Chromium Mouse Transcriptome Probe Set v1.0.1 mm10 with custom probes appended to the reference files using the Cell Ranger pipeline (cellranger-7.1.0) to generate gene-barcode matrices. These matrices were imported into Seurat (v4.1.0, R 4.1.3) for downstream analysis. Raw data was loaded into Seurat using the Read10X_h5 function. We filtered out cells with less than 200 genes, greater than 6000 genes, less than 500 reads or greater than 10% mitochondrial reads to remove low-quality cells. The data was normalized using the NormalizeData function with default parameters. The top 2000 highly variable features were identified using the FindVariableFeatures function and the data was scaled and centered using the ScaleData function. Principal component analysis (PCA) was performed using the RunPCA function. Clustering was conducted using the FindNeighbors and FindClusters functions with a resolution parameter set to 0.5. UMAP (Uniform Manifold Approximation and Projection) was used for visualization. $CD45^+$ immune cells were identified based on cell markers and clustered separately. Differential expression between clusters was performed using the FindMarkers function with a threshold of adjusted p-value = 0.25. Gene expression signature scores were generated using the AddModuleScore function with the default settings.

Tumor flow cytometry analysis

EMT6 tumors were implanted and treated as described above at 6e7 CFU/mouse and dissociated into single cell suspension using the Mouse Tumor Dissociation Kit (Miltenyi Biotec, 130-096-730). Cells were stained with live-dead dye (Thermo, L34976) followed by Fc Block (Biolegend, 156604), and pre-conjugated antibodies were incubated with samples for 30 minutes. For intracellular markers, cells were permeabilized (Thermo, 00-5523-00) followed by antibody staining for 30 minutes. Antibodies were washed off and samples were read on the flow cytometer.

All antibodies in the staining panels were purchased from BioLegend (Antigen, Cat#): CD45, #103125; I-A/I-E, #107635; Ly6G, #127639; CD11c, #117339; CD86, #105043; CD11b, #101219; CD206, #141705; F4/80, #123145; Ly6C, #128017; Ki-67, #652405; CD4, #100553; PD-1, #135219; CD69, #104541; CD3, #100231; FoxP3, #126405; OX40, #119409; Lag3, #125223; CD8, #100721; 41BB, #106109; CD73, #127215; CD44, #103049; CD127, #158203; CCR7, #120121; CD62L, #104411; CD19, #115507; CD39, #143811; F4/80, #123115.

Cells were gated using live-dead dye and CD45 into singlet live immune cells. Singlet live immune cells were further gated into T-cells using CD3, myeloid cells using CD11b, macrophages using F4/80, DCs using CD11b/c, neutrophils using Ly6G and T-cell subsets using CD4/8. For clustering analysis, the mean fluorescence intensities (MFIs) of $CD45^+$ immune cells were then biexponentially transformed. To correct for non-specific background signals, fluorescence-minus-one (FMO) controls were utilized. To integrate data across different samples, we utilized the Scanpy software package (version 1.9.5). Specifically, the Batch Balanced K-Nearest Neighbors (BBKNN) method was applied to account for batch effects and achieve a more uniform integration of the data. High-dimensional flow cytometry data were visualized by computing the UMAP. The Leiden clustering algorithm was then applied to the high-dimensional data to group cells into clusters, facilitating the identification of distinct cell populations based on the fluorescence intensity of specific markers. These intensities were scaled and transformed using the biexponential transformation. The proportions of each identified cell type were calculated and normalized per flow cytometry run to account for variability introduced by different experimental conditions. For the statistical analysis of fluorescence intensity data, FMO-corrected log10-transformed MFI values were used to stabilize variance and normalize the distribution. The Wilcoxon rank-sum test (for non-normally distributed data) was employed to compare changes in marker intensity and cell proportions between different experimental groups.

SUPPLEMENTARY REFERENCES

1. Toso JF, Gill VJ, Hwu P, Marincola FM, Restifo NP, Schwartzentruber DJ, Sherry RM, Topalian SL, Yang JC, Stock F, Freezer LJ, Morton KE, Seipp C, et al. Phase I study of the intravenous administration of attenuated Salmonella typhimurium to patients with metastatic melanoma. J Clin Oncol. 2002; 20:142–52. https://doi.org/10.1200/JCO.2002.20.1.142. [PubMed]
2. Horstmann JA, Lunelli M, Cazzola H, Heidemann J, Kühne C, Steffen P, Szefs S, Rossi C, Lokareddy RK, Wang C, Lemaire L, Hughes KT, Uetrecht C, et al. Methylation of Salmonella Typhimurium flagella promotes bacterial adhesion and host cell invasion. Nat Commun. 2020; 11:2013. https://doi.org/10.1038/s41467-020-15738-3. [PubMed]
3. Becerra-Báez EI, Meza-Toledo SE, Muñoz-López P, Flores-Martínez LF, Fraga-Pérez K, Magaño-Bocanegra KJ, Juárez-Hernández U, Mateos-Chávez AA, Luria-Pérez R. Recombinant Attenuated *Salmonella enterica* as a Delivery System of Heterologous Molecules in Cancer Therapy. Cancers (Basel). 2022; 14:4224. https://doi.org/10.3390/cancers14174224. [PubMed]
4. Sokaribo AS, Hansen EG, McCarthy M, Desin TS, Waldner LL, MacKenzie KD, Mutwiri G Jr, Herman NJ, Herman DJ, Wang Y, White AP. Metabolic Activation of CsgD in the Regulation of *Salmonella* Biofilms. Microorganisms. 2020; 8:964. https://doi.org/10.3390/microorganisms8070964. [PubMed]
5. Clairmont C, Lee KC, Pike J, Ittensohn M, Low KB, Pawelek J, Bermudes D, Brecher SM, Margitich D, Turnier J, Li Z, Luo X, King I, Zheng LM. Biodistribution and genetic stability of the novel antitumor agent VNP20009, a genetically modified strain of Salmonella typhimurium. J Infect Dis. 2000; 181:1996–2002. https://doi.org/10.1086/315497. [PubMed]
6. Ohta A. A Metabolic Immune Checkpoint: Adenosine in Tumor Microenvironment. Front Immunol. 2016; 7:109. https://doi.org/10.3389/fimmu.2016.00109. [PubMed]
7. Hajjar AM, Ernst RK, Tsai JH, Wilson CB, Miller SI. Human Toll-like receptor 4 recognizes host-specific LPS modifications. Nat Immunol. 2002; 3:354–59. https://doi.org/10.1038/ni777. [PubMed]
8. Luo X, Li Z, Lin S, Le T, Ittensohn M, Bermudes D, Runyab JD, Shen SY, Chen J, King IC, Zheng LM. Antitumor effect of VNP20009, an attenuated Salmonella, in murine tumor models. Oncol Res. 2001; 12:501–8. https://doi.org/10.3727/096504001108747512. [PubMed]
9. Alexander-Floyd J, Bass AR, Harberts EM, Grubaugh D, Buxbaum JD, Brodsky IE, Ernst RK, Shin S. Lipid A Variants Activate Human TLR4 and the Noncanonical Inflammasome Differently and Require the Core Oligosaccharide for Inflammasome Activation. Infect Immun. 2022; 90:e0020822. https://doi.org/10.1128/iai.00208-22. [PubMed]
10. Galán JE, Nakayama K, Curtiss R 3rd. Cloning and characterization of the asd gene of Salmonella typhimurium: use in stable maintenance of recombinant plasmids in Salmonella vaccine strains. Gene. 1990; 94:29–35. https://doi.org/10.1016/0378-1119(90)90464-3. [PubMed]
11. Kullas AL, McClelland M, Yang HJ, Tam JW, Torres A, Porwollik S, Mena P, McPhee JB, Bogomolnaya L, Andrews-Polymenis H, van der Velden AW. L-asparaginase II produced by Salmonella typhimurium inhibits T cell responses and mediates virulence. Cell Host Microbe. 2012; 12:791–98. https://doi.org/10.1016/j.chom.2012.10.018. [PubMed]
12. Lee KC, Zheng L-M, Luo X, Clairmont C, Fischer J, Margitich D, Turnier J, Almassian B, Bermudes D, King I. Comparative Evaluation of the Acute Toxic Effects in Monkeys, Pigs and Mice of a Genetically Engineered Salmonella Strain (VNP20009) Being Developed as an Antitumor Agent. Int J Toxicol. 2000; 19: 19–25. https://doi.org/10.1080/109158100225006.
13. Rubinstein MP, Kovar M, Purton JF, Cho JH, Boyman O, Surh CD, Sprent J. Converting IL-15 to a superagonist by binding to soluble IL-15R{alpha}. Proc Natl Acad Sci U S A. 2006; 103:9166–71. https://doi.org/10.1073/pnas.0600240103. [PubMed]
14. de Oliveira Mann CC, Orzalli MH, King DS, Kagan JC, Lee ASY, Kranzusch PJ. Modular Architecture of the STING

C-Terminal Tail Allows Interferon and NF-κB Signaling Adaptation. Cell Rep. 2019; 27:1165–75.e5. https://doi.org/10.1016/j.celrep.2019.03.098. [PubMed]

15. Konno H, Chinn IK, Hong D, Orange JS, Lupski JR, Mendoza A, Pedroza LA, Barber GN. Pro-inflammation Associated with a Gain-of-Function Mutation (R284S) in the Innate Immune Sensor STING. Cell Rep. 2018; 23:1112–23. https://doi.org/10.1016/j.celrep.2018.03.115. [PubMed]
16. Hansen AL, Mukai K, Schopfer FJ, Taguchi T, Holm CK. STING palmitoylation as a therapeutic target. Cell Mol Immunol. 2019; 16:236–41. https://doi.org/10.1038/s41423-019-0205-5. [PubMed]
17. Miao EA, Andersen-Nissen E, Warren SE, Aderem A. TLR5 and Ipaf: dual sensors of bacterial flagellin in the innate immune system. Semin Immunopathol. 2007; 29:275–88. https://doi.org/10.1007/s00281-007-0078-z. [PubMed]
18. Crull K, Bumann D, Weiss S. Influence of infection route and virulence factors on colonization of solid tumors by Salmonella enterica serovar Typhimurium. FEMS Immunol Med Microbiol. 2011; 62:75–83. https://doi.org/10.1111/j.1574-695X.2011.00790.x. [PubMed]
19. Miao EA, Alpuche-Aranda CM, Dors M, Clark AE, Bader MW, Miller SI, Aderem A. Cytoplasmic flagellin activates caspase-1 and secretion of interleukin 1beta via Ipaf. Nat Immunol. 2006; 7:569–75. https://doi.org/10.1038/ni1344. [PubMed]
20. Kawasaki K, Ernst RK, Miller SI. 3-O-deacylation of lipid A by PagL, a PhoP/PhoQ-regulated deacylase of Salmonella typhimurium, modulates signaling through Toll-like receptor 4. J Biol Chem. 2004; 279:20044–48. https://doi.org/10.1074/jbc.M401275200. [PubMed]
21. Batool T, Makky EA, Jalal M, Yusoff MM. A Comprehensive Review on L-Asparaginase and Its Applications. Appl Biochem Biotechnol. 2016; 178:900–23. https://doi.org/10.1007/s12010-015-1917-3. [PubMed]
22. Grantcharova N, Peters V, Monteiro C, Zakikhany K, Römling U. Bistable expression of CsgD in biofilm development of Salmonella enterica serovar typhimurium. J Bacteriol. 2010; 192:456–66. https://doi.org/10.1128/JB.01826-08. [PubMed]

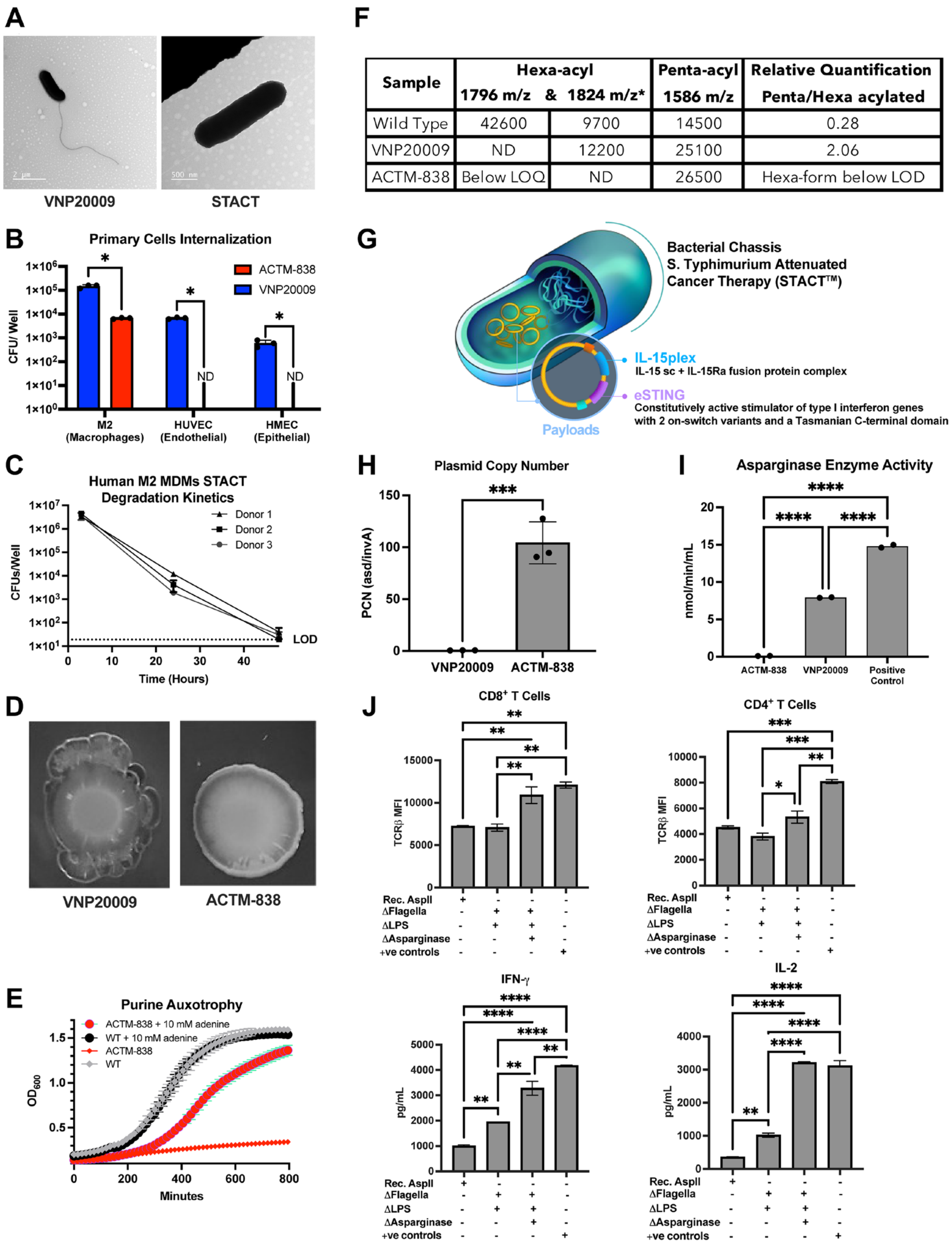

Supplementary Figure 1: Genetic engineering generates a morphologically distinct, auxotrophic salmonella typhimurium strain (STACT) with ability to encode payload carrying plasmids generating ACTM-838. (**A**) Electron microscopy of VNP20009 (left) and STACT chassis (right) shows VNP20009 expresses a flagellum while STACT chassis does not. (**B**) Flagella-deficient STACT (ACTM-838) is only internalized by primary human M2 phagocytic macrophages, and not HUVEC or HMEC, exhibiting myeloid uptake specificity. VNP20009 is internalized into all primary 3 cell types showing lack of cell type specificity. Statistical significance determined by unpaired *t*-test, $^{*}p < 0.01$. (**C**) ACTM-838 internalization in primary human M2 MDMs measured by CFU plating over time shows ACTM-838 gets degraded intracellularly. (**D**) ACTM-838 lacks the red, dry and rough phenotype (rdar) where darker colors and ridged texture depict biofilm formation made by VNP20009. (**E**) ACTM-838 requires purine i.e. adenine supplementation for growth, whereas WT *S.* Typhimurium can grow without it. (**F**) Relative quantitation of average peak intensities of lipid A ions in acid-hydrolyzed bacterial extracts, showing ACTM-838 exhibits only penta-acylated LPS unlike VNP20009 and WT which express both penta- and hexa-acylated LPS. (**G**) Schematic representation of ACTM-838, a STACT chassis strain carrying plasmids encoding 2 genetically engineered payloads IL-15 bound to IL-15Ra (IL-15plex) and a constitutively active variant of STING with a Tasmanian CTT (eSTING). (**H**) ACTM-838 payload plasmid is maintained *in vitro*, producing 100 plasmid copies per bacterium, while VNP20009 without a plasmid does not. Plasmid copy number (PCN) was measured by assessing expression of *asd* gene on plasmid versus *invA* gene on bacterial genome. Statistical significance determined by unpaired *t*-test, $^{***}p < 0.001$. (**I**, **J**) ACTM-838 is unable to hydrolyze extracellular asparagine to aspartic acid (due to deleted asparaginase enzyme) unlike VNP20009, restoring TCRβ expression in $CD4^{+}$ and $CD8^{+}$ T-cells (top panels), and IFNγ and IL-2 secretion (bottom panels). Statistical significance was determined using two-way ANOVA followed by Tukey's multiple comparisons test, $^{*}p < 0.05$; $^{**}p < 0.01$; $^{***}p < 0.001$; $^{****}p < 0.0001$. Abbreviations: HUVEC: human vascular endothelial cells; HMECs: human mammary epithelial cells; ND: not detected; LOD: limit of detection; TCRβ: T-cell receptor beta; CTT: C-terminal tail.

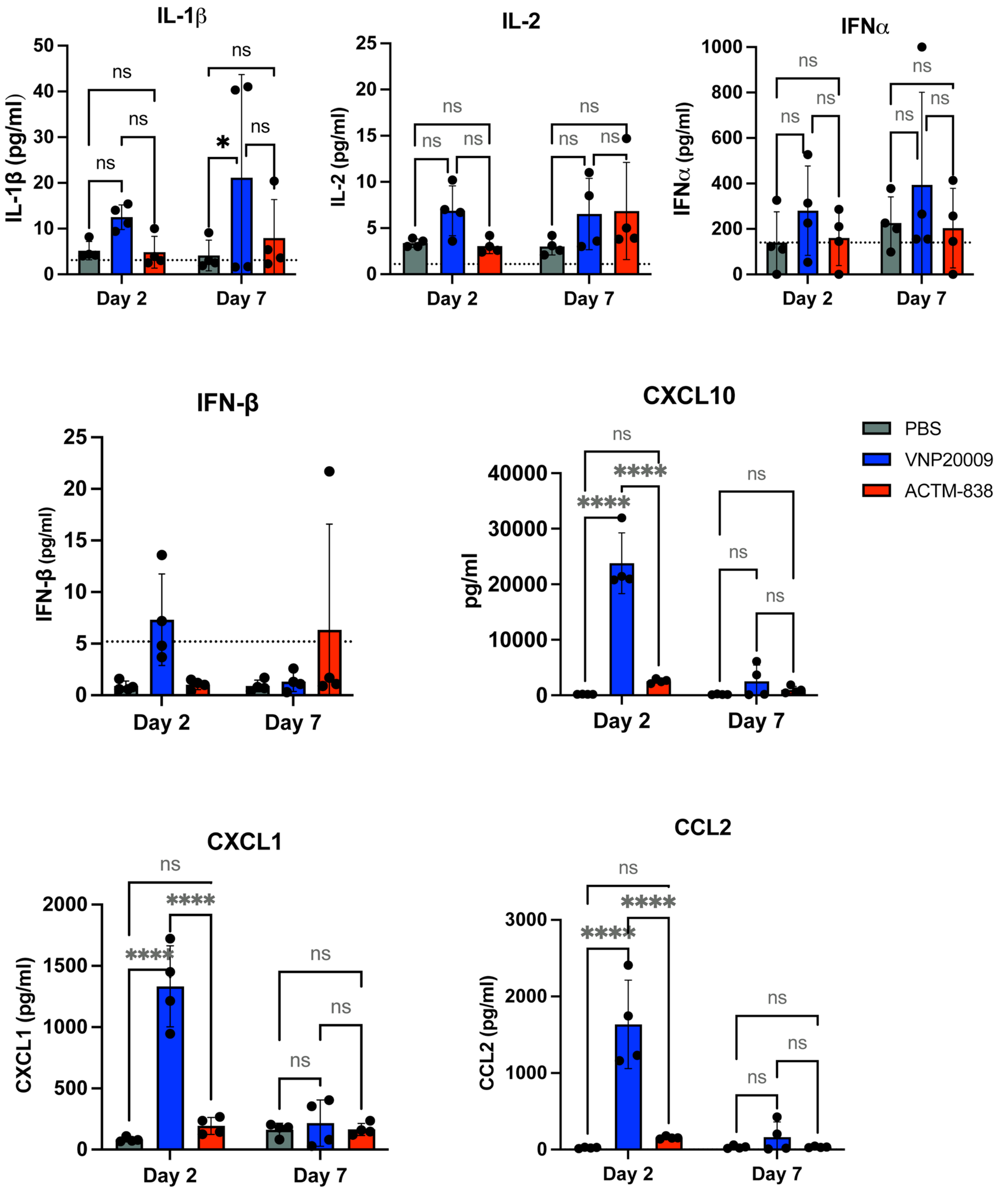

Supplementary Figure 2: Attenuated STACT strains including ACTM-838 exhibit lower levels of cytokines compared to parental strain VNP20009. ACTM-838 exhibit reduced systemic cytokines compared to VNP20009 in EMT6-tumor bearing BALB/c mice. EMT6 tumor-bearing BALB/c mice were administered 3e7 CFU/mouse of either ACTM-838 or VNP20009. Serum was collected at Day 2 and Day 7 post administration; cytokines were measured using Meso Scale Discovery. Statistical significance was determined using two-way ANOVA followed by Tukey's multiple comparisons test. Dotted line indicates lower limit of detection (LLOD) for given cytokine. $^{*}p < 0.05$; $^{**}p < 0.01$; $^{***}p < 0.0001$; $^{****}p < 0.00001$. Abbreviations: IL: interleukin; IFN: interferon; ns: not significant.

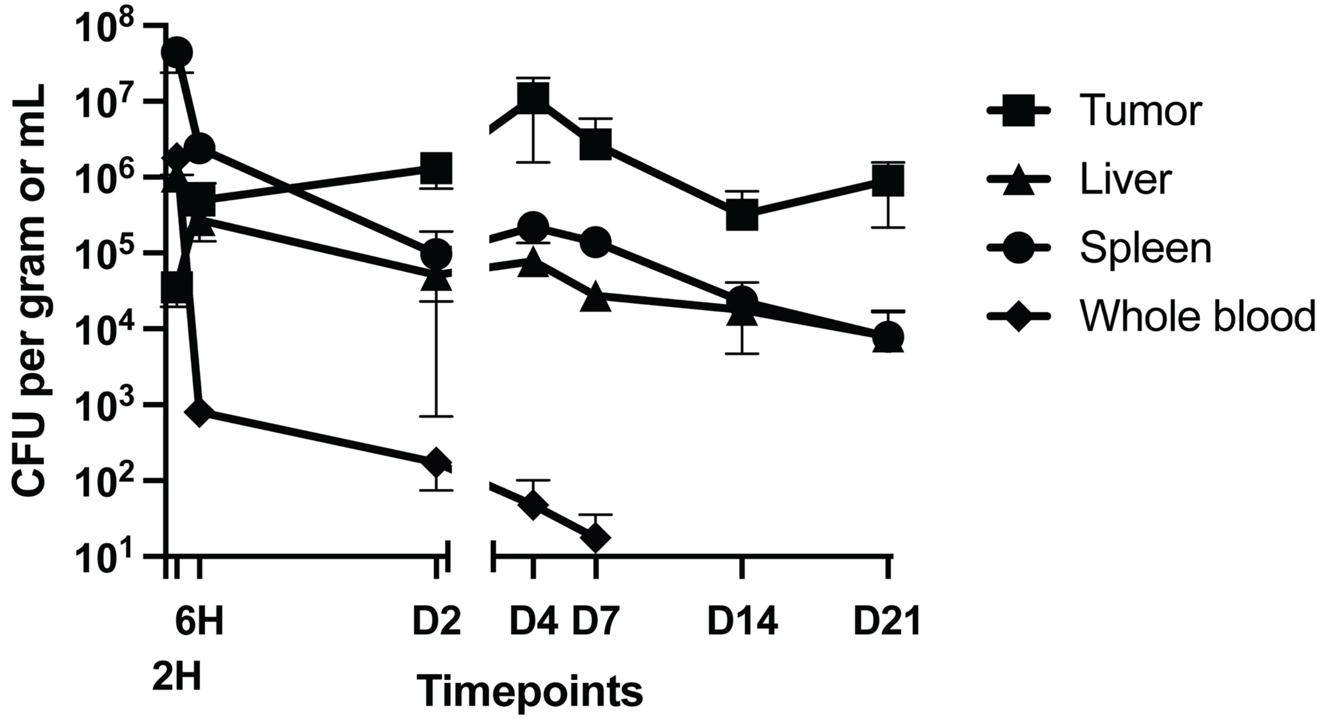

Supplementary Figure 3: Biodistribution of ACTM-838 in EMT6 tumor-bearing mice. Tissue biodistribution kinetics in tumor, liver, spleen and blood over time in EMT6-tumor bearing mice treated with ACTM-838 (3.8e7 CFU/mouse, *n* = 5 per timepoint) normalized for the weight of the tissue (per gram) and volume of blood (per mL). Note, this data is the same source data as Figure 1B, but plotted as CFU/g of tissue.

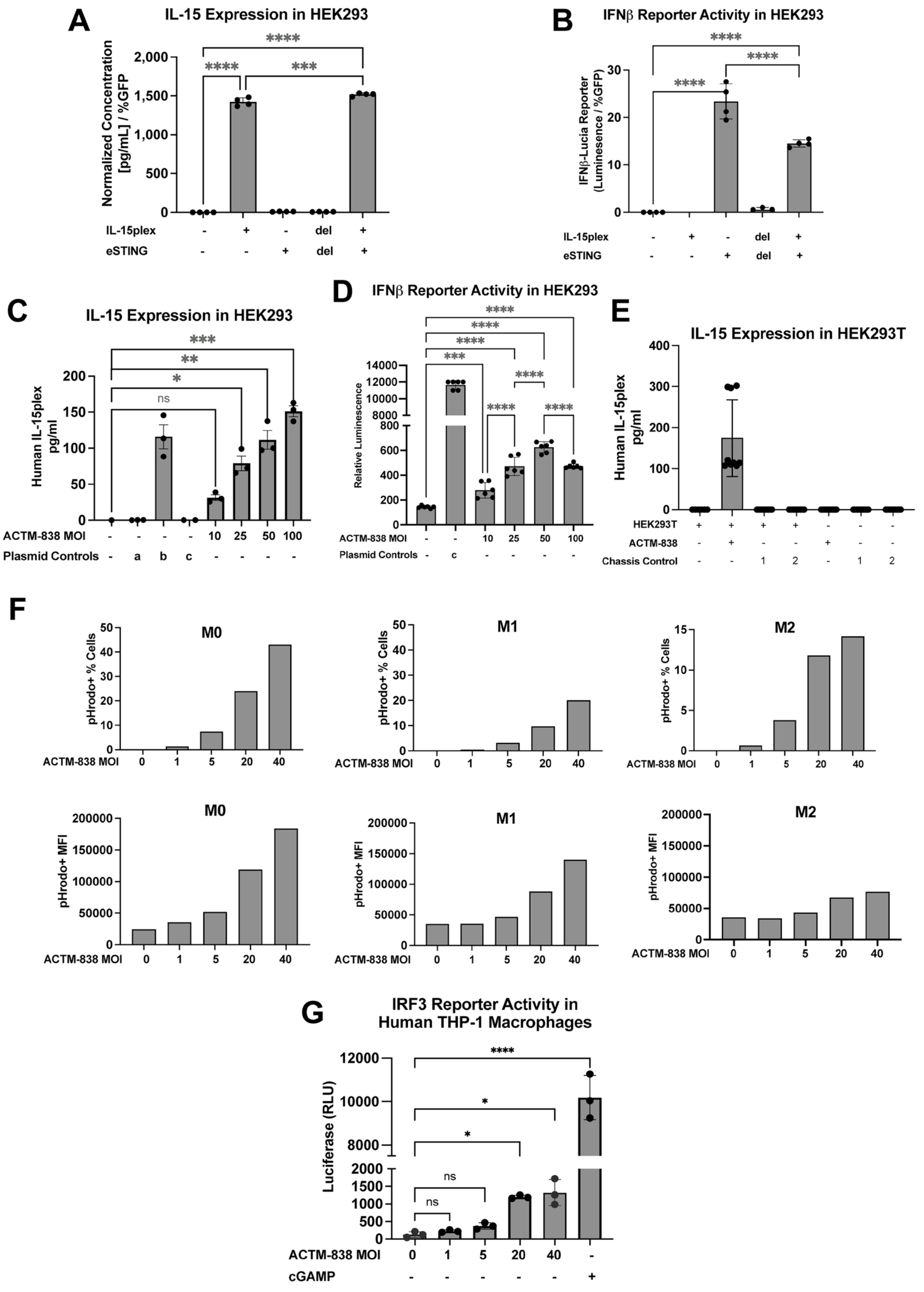

Supplementary Figure 4: ACTM-838 encodes payload plasmids leading to expression of IL-15plex and eSTING activity in target human cell types *in vitro*. (**A**, **B**) IL-15plex protein secretion and IFNβ luciferase reporter activity detected via ELISA in HEK293 Dual-Null IFNβ Lucia reporter cells with transfection of plasmids containing eSTING, hIL-15plex, truncated IL-15Ra with deleted CTT eSTING or hIL-15plex + eSTING (ACTM-838). (**C**, **D**) Expression of ACTM-838 encoded IL-15plex and eSTING payloads leads to dose dependent IL-15 secretion and IFNβ reporter activity in target HEK293 Dual-Null cells respectively. Negative controls, non-functional IL-15sushi (a) and IL-15sushi + eSTING with deleted CTT (c) did not exhibit any IL-15plex secretion, while plasmid IL15plex+eSTING (c – positive control) showed IL-15plex high expression and IFNβ reporter activity. (**E**) Supernatant from HEK293T cells treated with ACTM-838, chassis control 1 (STST-490, STACT strain without plasmid payload), chassis control 2 (STST-347, STACT strain delivering inactive IL-15plex and inactive STING), or supernatant from bacteria-only conditions were analyzed for IL-15plex expression. IL-15plex was only detected in conditions in which ACTM-838 was delivered to HEK293T cells. (**F**) pHrodo labeled ACTM-838 exhibits dose-dependent increase in lysosomal uptake (denoted by pHrodo dye positivity) in M0-, M1- and M2-like differentiated human THP-1 macrophages at 90 minutes post-treatment. (**G**) Dose-dependent eSTING-mediated IFNβ luciferase reporter expression at 48 hours post-ACTM-838 treatment. cGAMP is used as positive control. Data are plotted and presented as mean ± SD and analyzed via one-way ANOVA with multiple comparisons; $p > 0.05$ = not significant (ns), $^{*}p < 0.05$; $^{***}p < 0.001$. Abbreviations: ELISA: enzyme linked immunosorbent assay; GFP: green fluorescent protein; HEK: human embryonic kidney; IL: interleukin; IFN: interferon; MOI: multiplicity of infection; PBS: phosphate buffered saline.

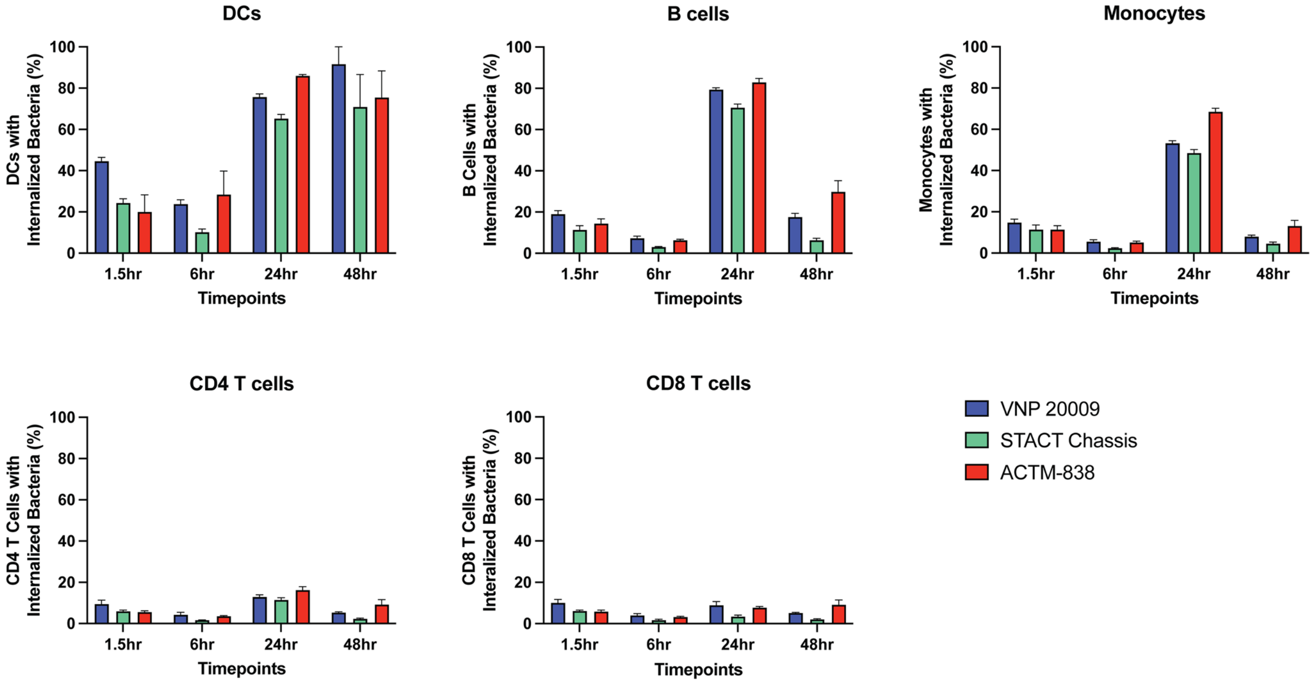

Supplementary Figure 5: ACTM-838 exhibits phagocytosis-mediated myeloid specific cellular internalization in healthy donor PBMCs. Flow cytometry analysis of human PBMCs after treatment with pHrodo-labeled ACTM-838, VNP-20009, or control STACT at various timepoints. Internalized bacteria were measured by flow cytometry using pHrodo denoting bacteria in acidic lysosomes within the phagocytic cells. Data is plotted and presented as mean ± SEM. Abbreviations: DC: dendritic cell; MOI: multiplicity of infection; PBMC: peripheral blood mononuclear cell; PBS: phosphate buffered saline.

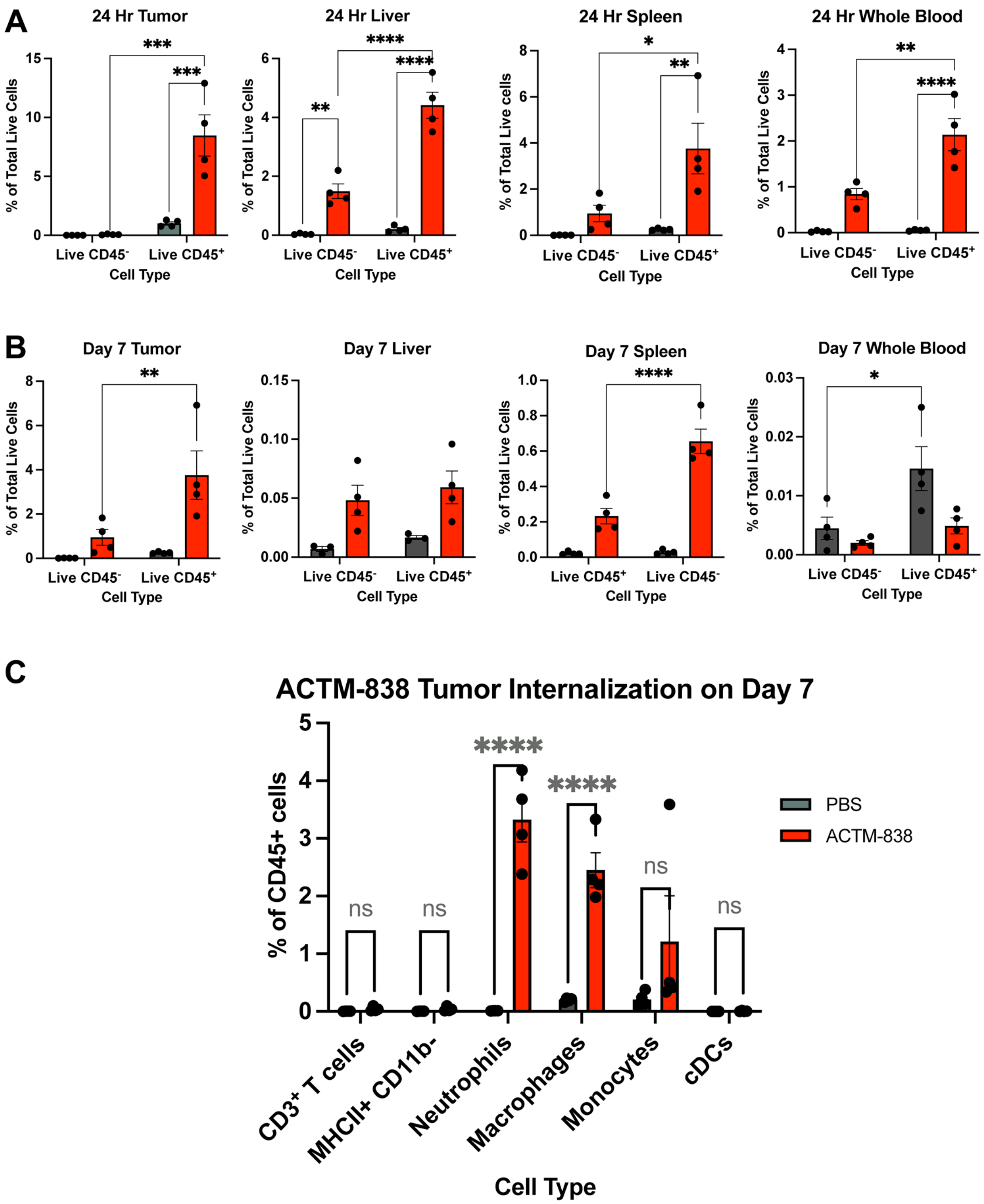

Supplementary Figure 6: ACTM-838 exhibits cellular uptake in CD45+ immune cells across tumor and healthy tissues in EMT6 tumor-bearing mice. (**A**, **B**) Internalization of ACTM-838 in Phagocytic APC Populations in EMT6 Tumor-Bearing BALB/c Mice at days 1 and 7 post-treatment using flow cytometry (ACTM-838 6e7 CFU/mouse). Cell lineage surface markers were used to identify distinct cell types and internalized ACTM-838 was identified via intracellular staining using an anti-LPS monoclonal antibody. (**C**) Cell subsets within CD45+ immune cells exhibiting internalization of ACTM-838 within immune subsets in the TME at Day 7 post treatment. $^{**}p < .001$; $^{****}p < .0001$. Abbreviations: CD: cluster of differentiation; CFU: colony-forming; PBS: phosphate buffered saline.

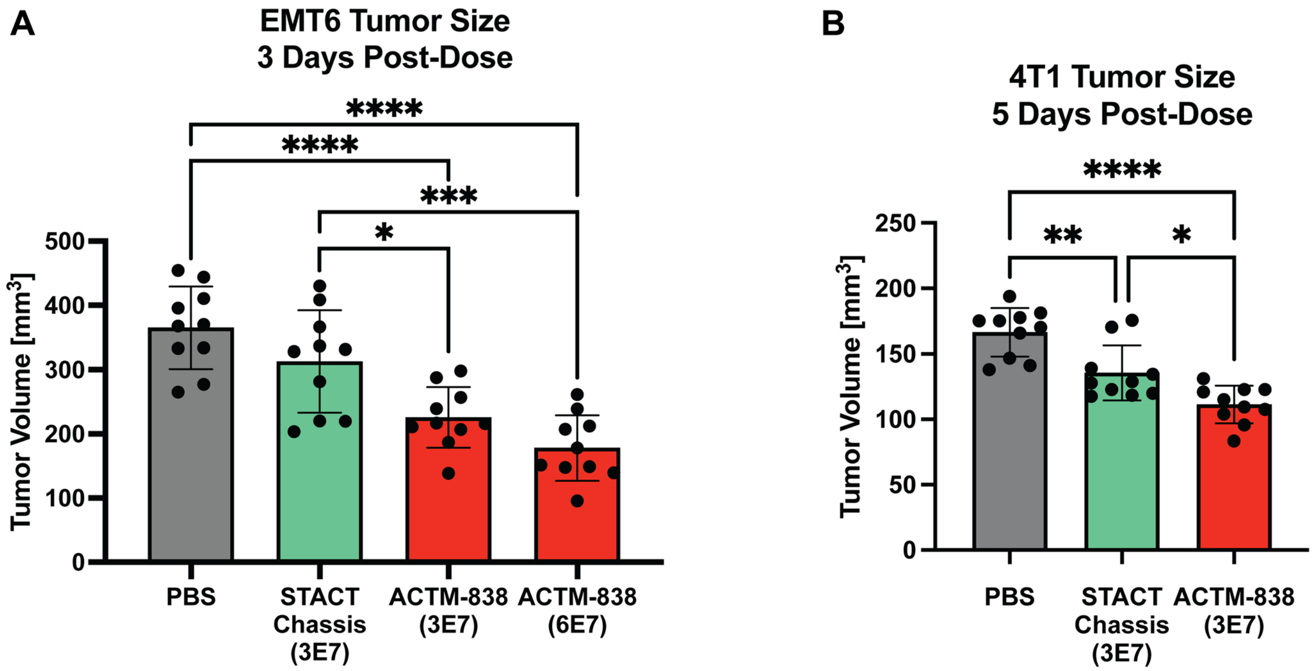

Supplementary Figure 7: Delivery of IL-15plex and eSTING by ACTM-838 increases anti-tumor efficacy over bacterial chassis alone. (**A**) EMT6- and (**B**) 4T1- tumor bearing BALB/c mice were dosed with PBS, ACTM-838 (3e7 CFU/mouse or 6e7 CFU/mouse, $n = 10$), or a bacterial chassis control (STST-490 at 3e7 CFU/mouse: STACT strain without payload plasmid and engineered with a restored, genomically integrated *asd* gene, $n = 10$). Administration of STACT chassis alone inhibits tumor growth while ACTM-838 delivery of IL-15plex and eSTING significantly increases the anti-tumor efficacy. EMT6: $^{****}p < 0.0001$, $^{***}p = 0.0001$, $^{*}p < 0.05$. 4T1: $^{****}p < 0.0001$, $^{**}p = 0.0020$, $^{*}p < 0.05$.

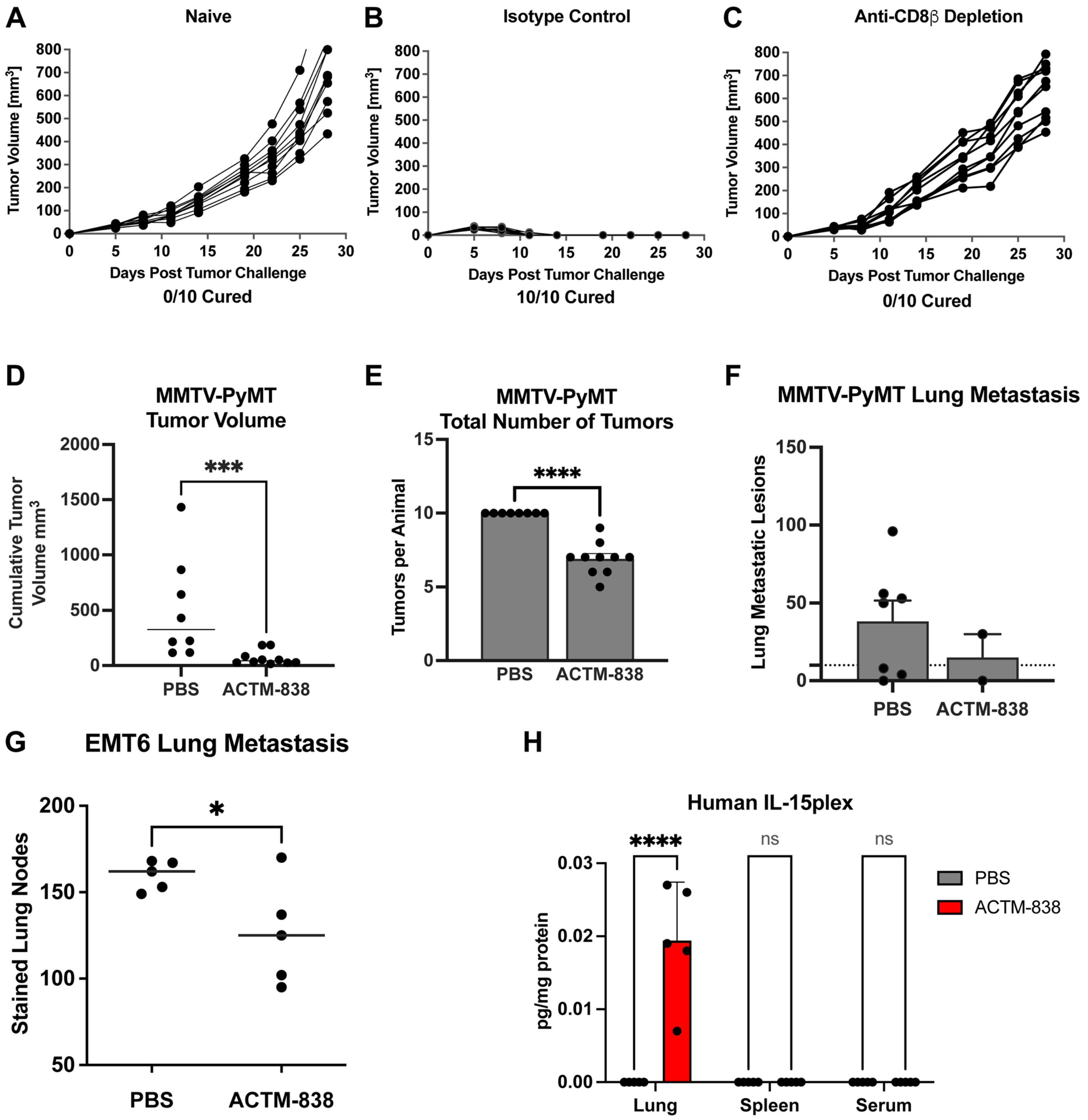

Supplementary Figure 8: ACTM-838 exhibits durable single agent efficacy across multiple syngeneic and GEMM models. ACTM-838-cured mice develop a durable anti-tumor CD8+ T-cell memory response. (**A**) Tumor-naïve BALB/c mice were implanted with 1e6 EMT6 cells in mammary fat pad. Previously ACTM-838 treated and cured mice received IP injections of either 100 mg IgG isotype control antibody (**B**) or CD8+ T-cell depleting antibody (**C**) 3 days prior to tumor re-challenge with 1e6 EMT6 cells implanted orthotopically into the contralateral mammary fat pad (Day 58 post-initial tumor implantation). Average circulating $CD8^+$ T-cells were 5.72% of total white blood cells for mice treated with the isotype control, and 0.48% for mice treated with anti-CD8b antibody. *N* = 10 per condition. (**D**, **E**) ACTM-838 (6e7 CFU/mouse) exhibited significantly reduced cumulative tumor volumes and total number of tumors per mouse on day 36 post treatment in MMTV-PyMT GEMM. (**F**) ACTM-838 treatment (6e7 CFU/mouse) reduced the number of lung metastasis in MMTV-PYMT GEMM. (**G**, **H**) Lung metastasis (nodule counts) and human IL-15plex protein levels in the EMT6 lung metastasis tumor model day 11 post dose (ACTM-838 dose at 6e7 CFU/mouse). $^{****}p < 0.0001$, $^{*}p < 0.05$; Abbreviation: ns: not significant.

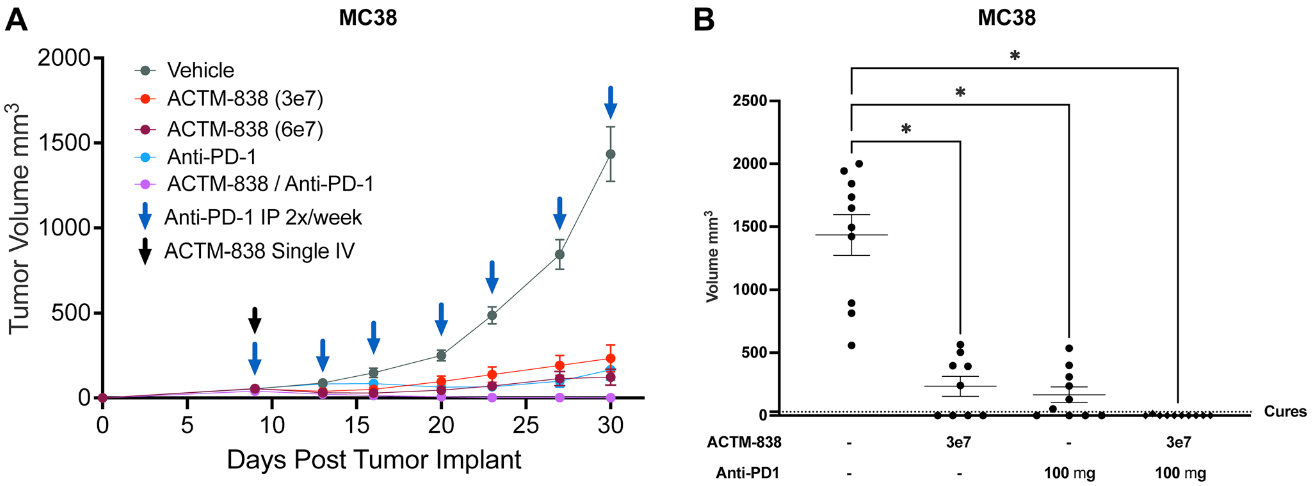

Supplementary Figure 9: ACTM-838 exhibits durable synergistic anti-tumor efficacy in combination with checkpoint blockade. (**A**, **B**) ACTM-838 shows synergistic combination efficacy with 100% durable remissions in MC38-tumor bearing mice over time and at endpoint (n = 10 per condition). Data are expressed tumor volume (mm^3) ± standard error of the mean (SEM). $^{***}p < 0.0001$ (Benjamini and Hochberg multiple comparisons test). Abbreviations: PBS: phosphate buffered saline; PD-1: programmed cell death protein 1; mm^3: cubic millimeter.

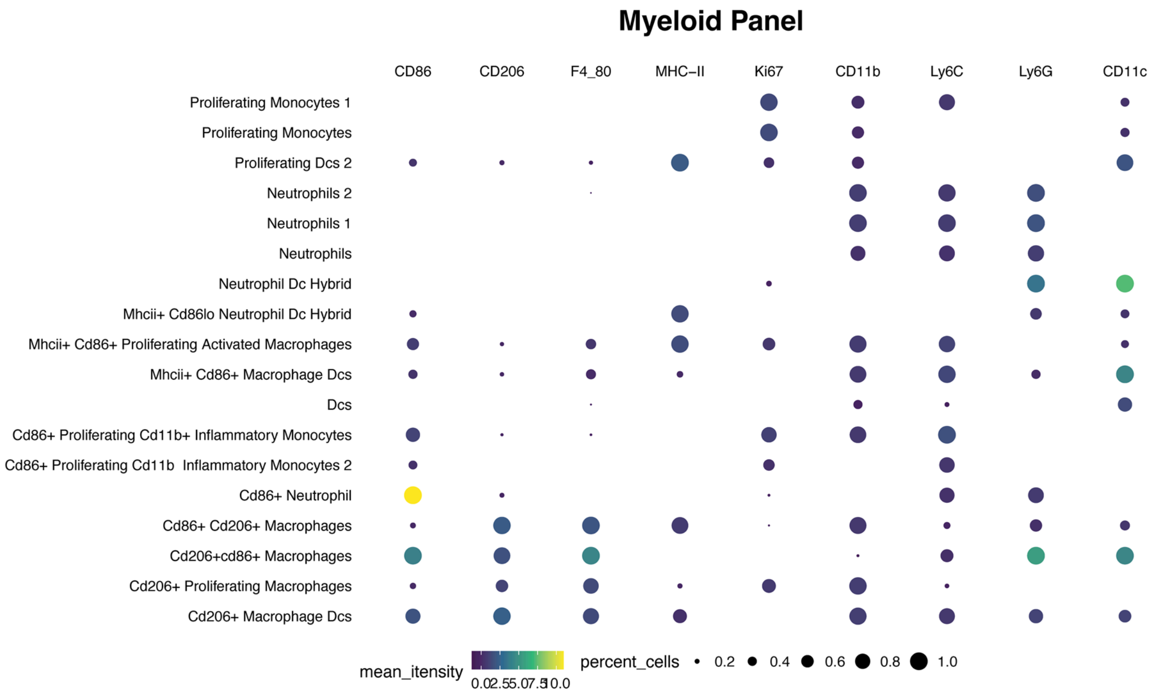

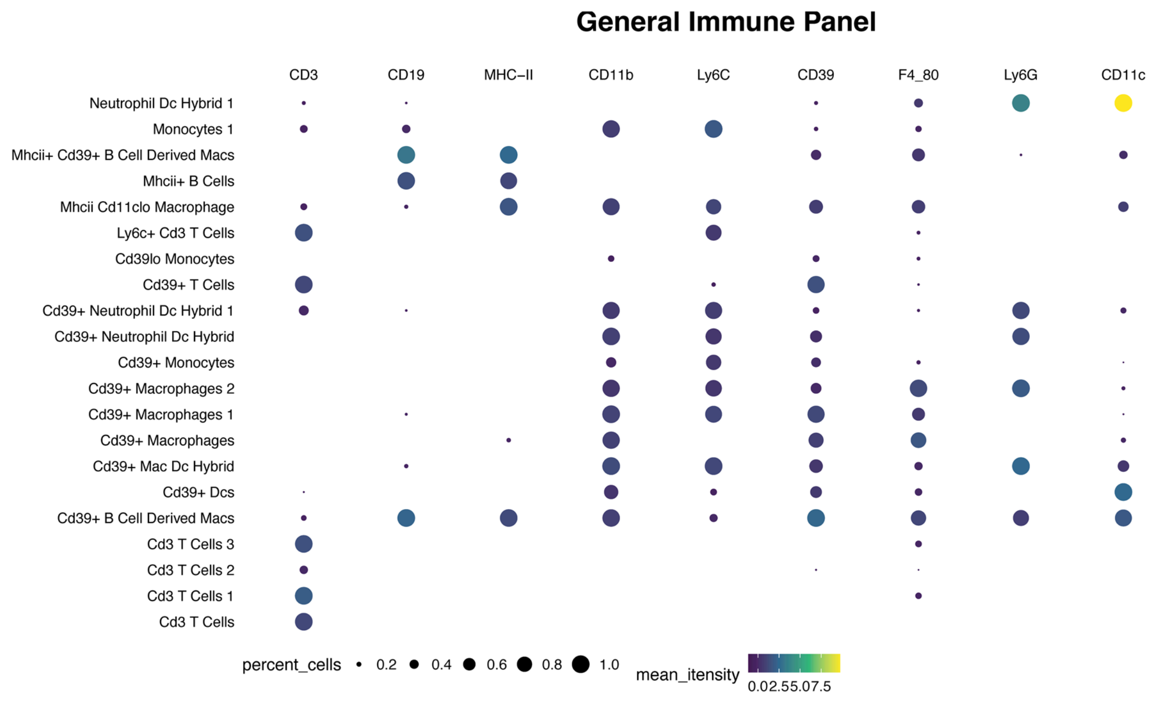

Supplementary Figure 10: Markers identifying cell populations in single cell flow cytometry analysis. Panels denote flow cytometry data from EMT6 tumor bearing mice at different timepoints (D0, D4, D7, D10) after ACTM-838 (6e7 CFU/mouse) and PBS treatments. EMT6 tumors were stained with 2 panels, each of the markers indicated on the top of each panel (See Methods). CD45+ immune cells were used in UMAP clustering analysis shown in the heatmap per panel. Y-axis denotes annotated cell populations based on canonical markers expressed on X-axis.

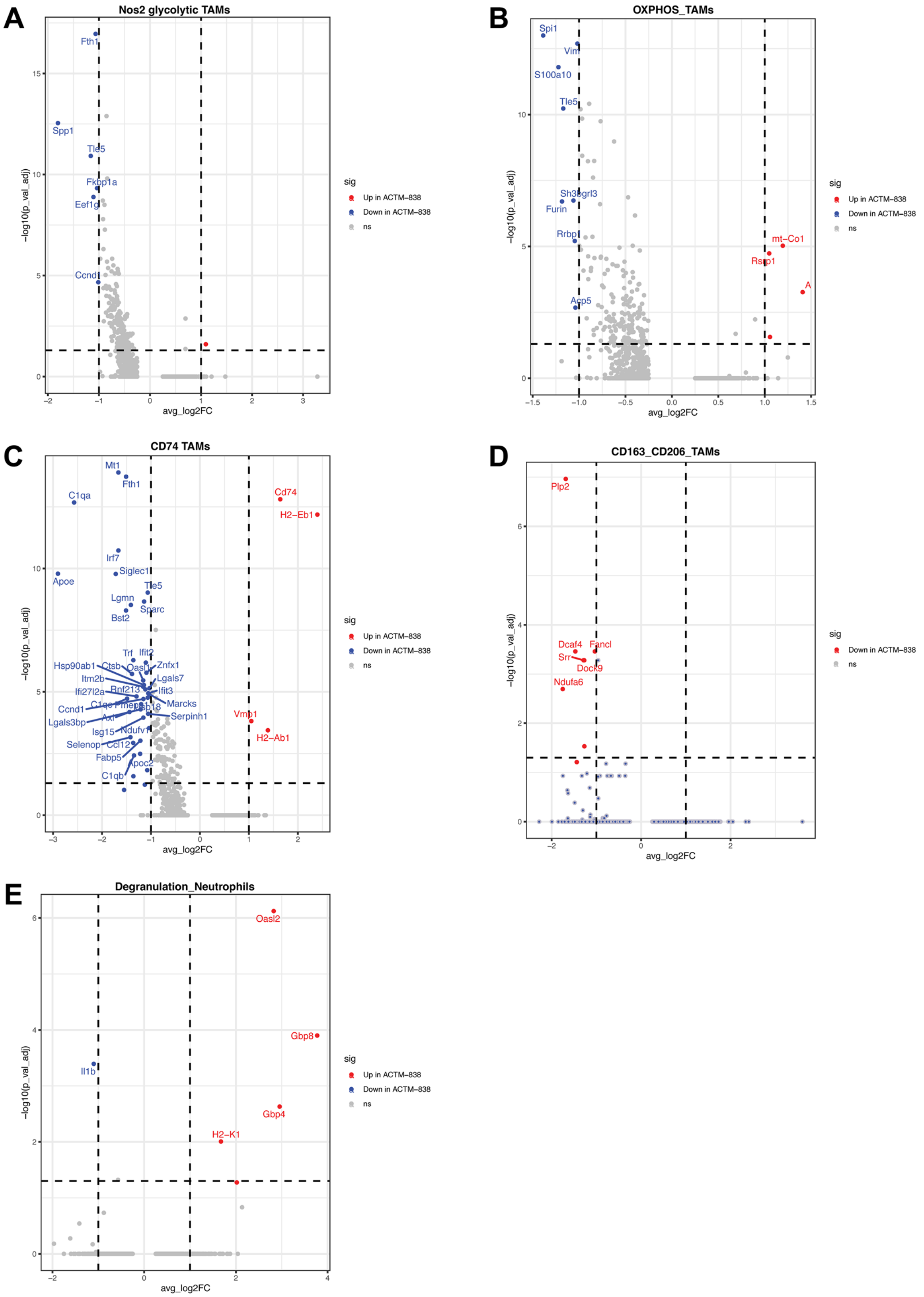

Supplementary Figure 11: Markers identifying cell populations in single cell RNAseq from EMT6 tumors treated with ACTM-838. Differential expression analysis of EMT6 tumor scRNAseq on day 7 post-ACTM-838 treatment (6e7 CFU/mouse, *n* = 2) for (**A**) Nos2 glycolytic TAMs, (**B**) OXPHOS TAMs, (**C**) CD74 TAMs, (**D**) CD163 CD206 TAMs, (**E**) degranulation neutrophils. Red and blue dots show significantly upregulated and downregulated genes in ACTM-838 vs. PBS. Abbreviation: ns: not significant.

Supplementary Table 1: STACT strain gene modifications

Product	Genotype	Functional Effect	Improved Safety/Activity	References
VNP20009	*purI*1757::Tn*10*	Purine/adenosine auxotrophy	• Tumor-specific enrichment<br>• Limited replication in healthy tissue	[5]
	Δ*msbB*	LPS surface coat modification	• Decreased TLR4 recognition<br>• Reduced immunosuppressive cytokines (TNF-α and IL-6)<br>• Inability to replicate in macrophages	[10,17]
STACT	Δ*msbB*	LPS surface coat modification	• Decreased TLR4 recognition<br>• Reduced immunosuppressive cytokines (TNF-α and IL-6)<br>• Inability to replicate in macrophages	[10,17]
	Δ*asd* + *asd* (on plasmid)	Plasmid maintenance	• Essential for plasmid delivery<br>• Plasmid maintenance via *asd* cassette	[10]
	Δ*fljB* + Δ*fliC*	Flagella knockout (Δ*fljB* + Δ*fliC*)	• Removes major inflammatory and immunosuppressive element<br>• Eliminates TLR5 recognition<br>• Eliminates flagella-induced inflammasome activation and pyroptotic cell death<br>• Inhibits stromal/tumor/endothelial cell infection	[17–19]
	Δ*pagP*	LPS surface coat modifications	• Removes major inflammatory and immunosuppressive element (reduced IL-6)<br>• Further decrease in TLR4 recognition via altered LPS acylation	[20]
	Δ*ansB*	L-asparaginase II knockout	• Eliminates extracellular L-asparaginase II activity<br>• Augments T-cell activity by removing immunosuppressive asparaginase	[11,21]
	Δ*csgD*	Removes: curli fimbriae; inflammatory CDNs; cellulose secretion	• Reduces TLR2-mediated immunosuppressive inflammation (curli fimbriae)<br>• Eliminates cellulose secretion/possible biofilm formation	[22]
	Δ*purl*	Complete deletion of *purI* gene; purine/adenosine auxotrophy	• Complete deletion of disrupted and inverted *purI* gene	[8]

The STACT strain was generated by engineering additional modifications into the parental VNP20009 strain. These additional modifications and their functional effects are listed in the above table. Abbreviations: CDN: cyclic dinucleotide; LPS: lipopolysaccharide; TLR: Toll-like receptor.

Supplementary Table 2: Antibiotic resistance of wildtype *S.* Typhimurium and ACTM-838

Antibiotic	Disc content (ug)	Interpretation criteria (zone diameter in mm)			WT *S.* Typhimurium zone diameter (mm)	ACTM-838 zone diameter (mm)
		Susceptible	Intermediate	Resistant		
Kanamycin	30	≥17	14–16	≤13	22	22
Streptomycin	10	≥23	20–22	≤19	12	9
Chloramphenicol	30	≥18	14–17	≤13	26	31
Ciprofloxacin	5	≥15	12–14	≤11	36	37
Meropenem	10	≥26	22–25	≤21	32	36
Sulphamethoxazole/ Trimethoprim	23.75/1.25	≥16	11–15	≤10	26	31
Aztreonam	30	≥15	12–14	≤11	29	38
Ceftriaxone	30	≥18	13–17	≤12	32	28
Piperacillin/ Tazobactam	100/10	≥25	21–24	≤20	28	30
Azithromycin	15	≥23	20–22	≤19	23	25

Sensitivity of ACTM-838 and wildtype S. Typhimurium to a panel of antibiotics were assessed using antibiotic testing discs. Interpretation criteria from the Clinical and Laboratory Standards Institute (CLSI) standards for Enterobacterales were utilized in the analysis of this data.

Supplementary Table 3: Biodistribution, shedding and blood pharmacokinetics ACTM-838 in EMT6 orthotopic tumor-bearing BALB/c mice

Organ	Measured CFUs						
	2 hours	6 hours	Day 2	Day 4	Day 7	Day 14	Day 21
Tumor	1,200 ± 560	68,000 ± 34,118	980,000 ± 833,907	660,000 ± 522,762	76,000 ± 90,510	101,200 ± 442,455	1,020,000 ± 658,089
Spleen	4,544,000 ± 1,403,596	278,000 ± 31,222	9,000 ± 18,766	57,800 ± 16,904	61,600 ± 8,807	15,400 ± 8,616	1,400 ± 3,333
Liver	282,200 ± 32,786	61,400 ± 20,432	8,000 ± 5,242	10,000 ± 1,220	4,400 ± 895	2,580 ± 1,653	820 ± 2,026
Kidney	NA	3,500 ± 198	160,000 ± 104,828	340 ± 117	180 ± 108	60 ± 40	0 ± 22
Heart	NA	4,760 ± 1,599	640 ± 1,151	620 ± 103	560 ± 253	100 ± 163	100 ± 27
Thymus	NA	300 ± 527	320 ± 304	80 ± 535	560 ± 253	320 ± 291	40 ± 44
Mesenteric lymph node	NA	140 ± 180	120 ± 194	560 ± 1279	260 ± 246	120 ± 156	0 ± 18
Intestine	NA	NA	0 ± 0	0 ± 0	0 ± 0	0 ± 0	0 ± 0
Muscle	NA	180 ± 89	100 ± 59	0 ± 29	0 ± 18	0 ± 9	0 ± 27
Brain	NA	NA	20 ± 25	40 ± 187	0 ± 98	10 ± 12	0 ± 27
Lung	NA	4,600 ± 1120	2,460 ± 2,081	2,000 ± 528	1,520 ± 536	900 ± 505	280 ± 382
Bone marrow	NA	975 ± 120	863 ± 532	545 ± 233	778 ± 611	220 ± 147	20 ± 18
Ovaries	NA	1,240 ± 1137	200 ± 184	480 ± 464	240 ± 393	0 ± 11	0 ± 11
Tumor draining lymph node	NA	NA	40 ± 66	20 ± 35	0 ± 44	0 ± 45	0 ± 27
Whole blood	3,200,000 ± 1,442,221	840 ± 178	160 ± 100	30 ± 55	10 ± 21	0 ± 0	0 ± 0
Urine	0 ± 0	0 ± 0	0 ± 0	0 ± 0	0 ± 0	0 ± 0	0 ± 0
Feces	0 ± 0	0 ± 0	0 ± 0	0 ± 0	0 ± 0	0 ± 0	0 ± 0

ACTM-838 dose is 3.8e7 CFU/mL, $n = 5$ per timepoint. The limit of detection (LOD) for all tissues is 20 CFU/organ and for whole blood is 10 CFU/mL; The CFUs observed in the lung are likely due to tumor cells metastasizing to this tissue at this timepoint and bacterial colonization of the metastases. Values shown are median CFU ± standard deviation per organ. Bone marrow, blood and urine are shown as CFU/mL. NA: data not available due to tissues not collected at a given timepoint.
